# Bacterial profile and antimicrobial resistance patterns of bloodstream infections at Amhara Public Health Institute, Bahir Dar, Ethiopia

**DOI:** 10.1371/journal.pone.0354710

**Published:** 2026-07-27

**Authors:** Michael Getie, Wudu Tafere, Alem Tsega, Tsehaynesh Gebreyesus, Gizeaddis Belay, Hailu Getachew, Yosef Gashaw, Aimro Tadese, Tigist Birku, Tigabu Birhan, Alemayehu Abate, Teshiwal Deress, Gebremedhin Necho, Belay Bezabih

**Affiliations:** 1 Medical Microbiology, Amhara Public Health Institute, Bahir Dar, Ethiopia; 2 Medical Biotechnology, Amhara Public Health Institute, Bahir Dar, Ethiopia; 3 Department of Quality Assurance and Laboratory Management, School of Biomedical and Laboratory Sciences, College of Medicine and Health Sciences, University of Gondar, Gondar, Ethiopia; 4 Field Epidemiology, Amhara Public Health Institute, Bahir Dar, Ethiopia; Hawassa University College of Medicine and Health Sciences, ETHIOPIA

## Abstract

**Background:**

Managing bloodstream infections in resource-constrained regions like Ethiopia is challenging due to scarce blood culture surveillance data. To guide empirical therapy, this study determines the bacterial profiles and antimicrobial resistance patterns among patients with suspected bloodstream infections at the Amhara Public Health Institute.

**Methods:**

This retrospective study analyzed blood culture records from the Amhara Public Health Institute spanning January 1, 2020, to December 30, 2023. Blood samples were processed using standardized manual culture techniques in accordance with World Health Organization (WHO) protocols. Antimicrobial susceptibility testing was performed using the Kirby-Bauer disk diffusion method following Clinical and Laboratory Standards Institute (CLSI) guidelines. Statistical analysis was conducted using SPSS version 20, employing descriptive statistics and regression models, with statistical significance defined as p < 0.05.

**Results:**

True bacterial pathogens were isolated from 50.3% (N = 340) of the 676 patients, with Gram-negative bacteria predominating (61.2%, N = 208) over Gram-positive bacteria (38.8%, N = 132). Notably, ESKAPEE pathogens accounted for 96.5% (N = 328) of all isolates, led by *Klebsiella* spp. (25.9%, N = 88), *Enterococcus* spp. (20.9%, N = 71), and *S. aureus* (15.6%, N = 53). Among Gram-positive isolates, high resistance was observed against oxacillin (74.5%, N = 41), penicillin (72%, N = 36), and vancomycin (51.1%, N = 24). Gram-negative isolates exhibited critical resistance to national frontline therapeutics, including ampicillin (100%, N = 24), ceftriaxone (92.4%, N = 157), and trimethoprim-sulfamethoxazole (89.4%, N = 126). Overall, multidrug-resistant (MDR), extensively drug-resistant (XDR), and pandrug-resistant (PDR) profiles were detected in 43.8% (N = 149), 30.5% (N = 104) and 5.3% (N = 18) of isolates with similar distributions observed among ESKAPEE strains. Sex and age were the only independent predictors of culture positivity, with female sex reducing bloodstream infection odds by 44% (p = 0.002). Conversely, neonates (≤ 28 days) and young adults (15–24 years) had 4.7 times (p = 0.001) and 9.5-times (p = 0.001) higher odds compared to elderly patients.

**Conclusions:**

This study reveals a severe 50.3% (N = 340) bloodstream infection rate dominated by highly resistant Gram-negative and ESKAPEE pathogens, disproportionately affecting males, neonates, and young adults. Combating this threat requires immediate antibiotic stewardship, updated guidelines, and advanced laboratory testing (anaerobic culture, minimum inhibitory concentration, and molecular sequencing) to track and manage resistance.

## Background

Bloodstream infections (BSIs) are severe, life-threatening conditions primarily caused by bacteria, fungi, viruses, and protozoa can also invade the bloodstream [1]. Globally, they are associated with high morbidity, prolonged hospital stays, increased intensive care needs, and significant mortality rates ranging from 20% to 50%. In 2020, the World Health Organization (WHO) reported that sepsis a common complication of BSIs affects 49 million people and causes 11 million deaths annually, disproportionately affecting children [2,3]. Symptoms range from fever, chills, and altered mental status to severe complications like septic shock and multi-organ failure.

The prevalence of BSIs varies across geographic regions and healthcare settings, influenced by factors such as patient demographics, medical practices, antibiotic usage, and antimicrobial resistance (AMR) patterns [4]. Globally, bacteria are the most frequently isolated pathogens. In low- and middle-income countries (LMICs), the most common isolates belong to the ESKAPEE group (*Enterococcus* spp., *S. aureus, K. pneumoniae*, *A. baumannii*, *P. aeruginosa*, *Enterobacter* spp. and *E. coli*). These are recognized by the WHO as critical and high-priority pathogens due to their high virulence and ability to cause severe, costly complications [5]. Other common isolates include *S. viridans*, *S. pneumoniae*, *S. pyogenes* and Coagulase-negative staphylococci (CoNS), with fungal isolates being rarer.

Antimicrobial resistance (AMR) in bloodstream infections poses a severe and rapidly escalating threat to global public health. In 2019, drug-resistant infections contributed to an estimated 4.95 million deaths worldwide. Projections indicate that without immediate, coordinated intervention, annual AMR-related mortality could reach 10 million by 2050 [5]. Resource-limited regions, including Ethiopia, bear the brunt of this crisis, accounting for the vast majority of these AMR-associated deaths. WHO reports highlight critically high levels of resistance in BSI-causing pathogens, including Methicillin-Resistant *S. aureus* (MRSA), and 3rd-generation cephalosporin and carbapenem-resistant *K. pneumoniae*, *A. baumannii* and *E. coli* [6]. Infections caused by these multidrug-resistant (MDR) organisms severely limit therapeutic options, require expensive medications, and increase both hospital stays and mortality [6].

Managing BSIs in developing countries such as Ethiopia remains largely empirical, heavily relying on clinical judgment in the absence of routine bacterial culture and susceptibility testing. Studies indicate that BSI prevalence among suspected septicemia cases in Ethiopia is 30.66% [7], fluctuating between 4.0% and 41.5% depending on patient risk factors [8]. The mortality rate for BSIs is approximately 50% [9]. Since bacterial pathogens and their resistance profiles are geographically variable and change rapidly, ongoing blood culture surveillance is vital for effective clinical management [10]. Therefore, this study determines the bacterial profiles and antimicrobial resistance patterns among patients with suspected BSIs at the Amhara Public Health Institute.

## Materials and methods

### Study design, period, and study area

This four-year retrospective study analyzed 676 blood culture and antimicrobial susceptibility records from the Amhara Public Health Institute (APHI) bacteriology reference laboratory between January 1, 2020 and December 30, 2023. The institute’s fully accredited laboratory plays a vital role in managing infectious diseases for a population of over 28.54 million in north-western and north-central Ethiopia [11].

### Data collection

Laboratory blood culture records were accessed on January 6, 2025, and data were retrieved using a standardized data collection form. The extracted variables encompassed patient sex, age, bacterial growth status, isolated microbial species, and antimicrobial susceptibility profiles. For stratified analysis, patients were categorized into three age groups: (≤ 28 days), pediatrics (>28 days to 14 years), and adults (≥ 15 years). Records with missing or illegible data, as well as those identified as blood culture contaminants, were excluded from the final analysis.

#### Blood samples collection.

Blood samples were collected according to standardized manual blood culture methods following WHO recommendations [12]. The pre-analytical phase of blood culture procedures have a significant impact on the sensitivity, interpretation, and clinical relevance of blood cultures. The positivity of a blood culture depends on the volume of blood, number of blood culture sets and timing of blood cultures [13]. The site of venipuncture was properly disinfected with 70% alcohol followed by 2% tincture of iodine to avoid contamination of the blood culture with skin flora. Depending on institute manual blood culture collection standard operating procedure (SOP) adults, 10 ml per bottle (two aerobic bottles), pediatrics a maximum of 5 ml per bottle (two aerobic bottles) and for neonates 1 ml per bottle (two aerobic bottles) of blood sample was drawn within a 24-hour period via separate peripheral venipuncture prior to antibiotic administration [14].

#### Blood culture.

Following collection, blood samples were immediately transferred into blood culture bottles containing tryptone soy broth (TSB) at a standard 1:10 blood-to-broth ratio. Inoculated bottles were transported at room temperature to the laboratory within 30 minutes of collection. The bottles were then incubated at 35–37°C for up to seven days and inspected daily for macroscopic signs of microbial growth. Cultures displaying evidence of growth were sub cultured using manual blood culture systems [12].

#### Sub-culture of primary blood culture.

Positive blood cultures were sub cultured onto blood agar plates (BAP), MacConkey agar plates (MAP), and chocolate agar plates (CAP). The BAP and CAP media were incubated under microaerophilic conditions using a candle jar at 35–37°C for 48 hours, whereas MAP media were incubated aerobically at the same temperature for 24 hours. Bacterial identification was subsequently performed based on colony morphology, hemolysis patterns, Gram staining, and standard biochemical tests.

#### Bacteria identification.

Following the isolation of pure cultures from subculture plates, bacterial identification was guided by Gram stain results. Gram-negative bacilli were characterized using a panel of conventional biochemical assays, including triple sugar iron (TSI) agar fermentation, indole production, citrate utilization, urease activity, motility, lysine decarboxylase (LDC) and oxidase testing [15]. Gram-positive bacteria were identified using conventional biochemical assays, including catalase, coagulase, bile esculin agar and mannitol fermentation, in accordance with the Clinical and Laboratory Standards Institute (CLSI) 2024 guidelines [16,17]. *Candida* spp. were identified based on macroscopic features, including colony morphology, growth rate, and surface texture, and further confirmed via microscopic examination and the germ tube test [7].

### Antimicrobial susceptibility testing

Antimicrobial susceptibility testing (AST) was performed for each isolate on Mueller–Hinton agar using the standardized Kirby–Bauer disk diffusion method, in accordance with the Clinical and Laboratory Standards Institute (CLSI) 2024 (M100) guidelines [16]. Bacterial suspensions were prepared, adjusted, and inoculated onto the agar surface. Gram-positive isolates were tested against chloramphenicol (30 μg), cefoxitin (30 μg), penicillin (10 units), gentamicin (10 μg) and vancomycin (30 µg). Gram-negative isolates were evaluated using ampicillin (10 µg), amoxicillin-clavulanic acid (30 μg), ceftriaxone (30 μg), ceftazidime (30 μg), chloramphenicol (30 μg), ciprofloxacin (5 μg), gentamicin (10 μg), imipenem (10 μg), meropenem (10 μg), trimethoprim-sulfamethoxazole (1.25/23.75 μg), tobramycin (10 μg) and piperacillin-tazobactam (100/10 μg). These specific antimicrobial agents were selected based on their local availability and high prescription frequency for managing bloodstream infections in Ethiopia, particularly within the study area. Following overnight incubation, zones of inhibition were measured to categorize the isolates as susceptible, intermediate, or resistant. All antibiotic disks were sourced from Oxoid Ltd. (Basingstoke, Hampshire, UK).

### Definitions

Bacterial isolates were classified as multidrug-resistant (MDR), extensively drug-resistant (XDR), or pandrug-resistant (PDR) according to the criteria defined by Magiorakos et al [9].

- Multi-drug resistance: defined as non-susceptibility to at least one agent in three or more antimicrobial classes [9].
- Extensive-drug resistance: defined as non-susceptibility to at least one agent in all but two or fewer antimicrobial categories tested for a particular microorganism [9].
- Pan-drug resistance was defined as non-susceptibility all to agents in all antimicrobial classes for each bacterium in this study [9].
- Blood culture contamination: defined as the growth of common skin commensals in only a single blood culture set out of a series [18,19].
- Contaminant species: included *Diphtheroids* (excluding *Corynebacterium diphtheriae*), *Bacillus* spp. (excluding *Bacillus anthracis*), coagulase-negative *staphylococci* (CoNS), *Propionibacterium* spp, *Aerococcus* spp, *Micrococcus* spp. and *Streptococci viridans* regardless of the number of positive blood culture bottles [18,19].

### Quality control

Standard operating procedures (SOPs) for blood culture processing were strictly followed across all phases including sample collection, transportation, inoculation, incubation, and biochemical identification to ensure data accuracy and reliability. Media quality control was performed by randomly selecting 5% of each prepared batch and incubating it aerobically at 35–37°C for 24 hours to confirm sterility. Media performance was further validated using standard control strains prior to inoculation and susceptibility testing. Antibiotic disks were selected based on local availability in accordance with CLSI guidelines [16]. Quality control for organism identification and susceptibility testing was maintained using reference strains, including *E. coli* ATCC 25922, *S. aureus* ATCC 25923 and *P. aeruginosa* ATCC 27853. Finally, senior microbiologists verified the accuracy of all inoculation techniques, colony characterizations, zone measurements, and AST interpretations.

### Data analysis and interpretation

Statistical analysis was performed using SPSS version 20 for data entry, cleaning, coding, and modeling. Descriptive statistics, including frequencies and percentages, were calculated to summarize the study variables. To assess the strength of association between independent and dependent variables, binary logistic regression models were utilized. Variables demonstrating a *p*-value < 0.20 in the bivariate analysis were entered into the multivariate logistic regression model to control for confounding and identify independent associations. Statistical significance was evaluated using adjusted odds ratios (AOR) with corresponding 95% confidence intervals (CI). Finally, the data analyzed was presented using text, tables, and graphs.

### Ethical consideration

Ethical approval for this study was granted by the regional public health research Ethical Review Committee (ERC) (Ref: NOH/R/T/D/07/74). Furthermore, permission was obtained from the institute’s laboratory director and the head of the bacteriology reference laboratory. Because the study utilized secondary, routinely collected laboratory culture data, the requirement for patient informed consent was waived by the ERC. To ensure patient confidentiality, all personal identifiers were removed, and the dataset was analyzed entirely anonymously. All relevant data was included within the manuscript and supporting information files without restriction. The study was conducted in full compliance with the ethical principles of the Declaration of Helsinki.

## Results

### Socio- demographic and clinical characteristics of study participants

Between 2020 and 2023, a total of 817 blood culture records were retrieved; 141 were excluded as contaminants, leaving 676 records for the final analysis. The mean age of the participants was 15.86 years (SD ± 20.8 years; range: 1 day to 90 years). More than half of the participants were male (58.9%, N = 398). The most represented age groups were infants aged 29 days to 5 years (27.8%, N = 188), followed by neonates (**≤** 28 days) (23.8%, N = 161). Regarding clinical management settings, most of the study participants were treated in inpatient department (89.1%, N = 602) (Table 1).

**Table 1:** Socio-demographic and clinical characteristics of patients with suspected bloodstream infections at Amhara Public Health Institute, 2020-2023.

Variables	Category	Frequency (N = 676)	Percentage (%)
Sex	Male	398	58.9
	Female	278	41.1
Age group	**≤** 28 days	161	23.8
	29 days – 5 years	188	27.8
	6-14 years	79	11.7
	15-24 years	57	8.4
	25-54 years	139	20.6
	55-64 years	27	3.9
	≥ 65 years	25	3.7
Patient’s clinical setting	Inpatient department	602	89.1
	Outpatient department	74	10.9
Clinical history	Acute febrile illness	6	0.9
	Bloodstream infection	507	75
	Chronic heart failure	20	2.9
	Hospital-acquired infection	79	11.7
	Meningitis	19	2.8
	Rheumatoid arthritis	4	0.6
	Sever acute malnutrition	4	0.6
	Shock	4	0.6
	Surgical site infection	24	3.6
	Upper respiratory tract infection	5	0.7
	Urinary tract infection	4	0.6
Prior antimicrobial use	No	146	21.6
	Yes	530	78.4
Years of diagnosis	2020	181	26.8
	2021	259	38.3
	2022	209	30.9
	2023	27	3.9

Regarding clinical indications, many participants were investigated for suspected community-acquired bloodstream infections (75.0%, N = 507), followed by hospital-acquired infections (11.7%, N = 79) and surgical site infections (3.6%, N = 24). Notably, a high proportion of the study population (78.4%, N = 530) reported a history of prior antimicrobial use. In terms of temporal distribution, the highest sample volume was recorded in 2021, accounting for 38.3% (N = 259) of the total study population (Table 1).

### Bacteriological profiles of bloodstream infection suspected patients

**Fig 1:**
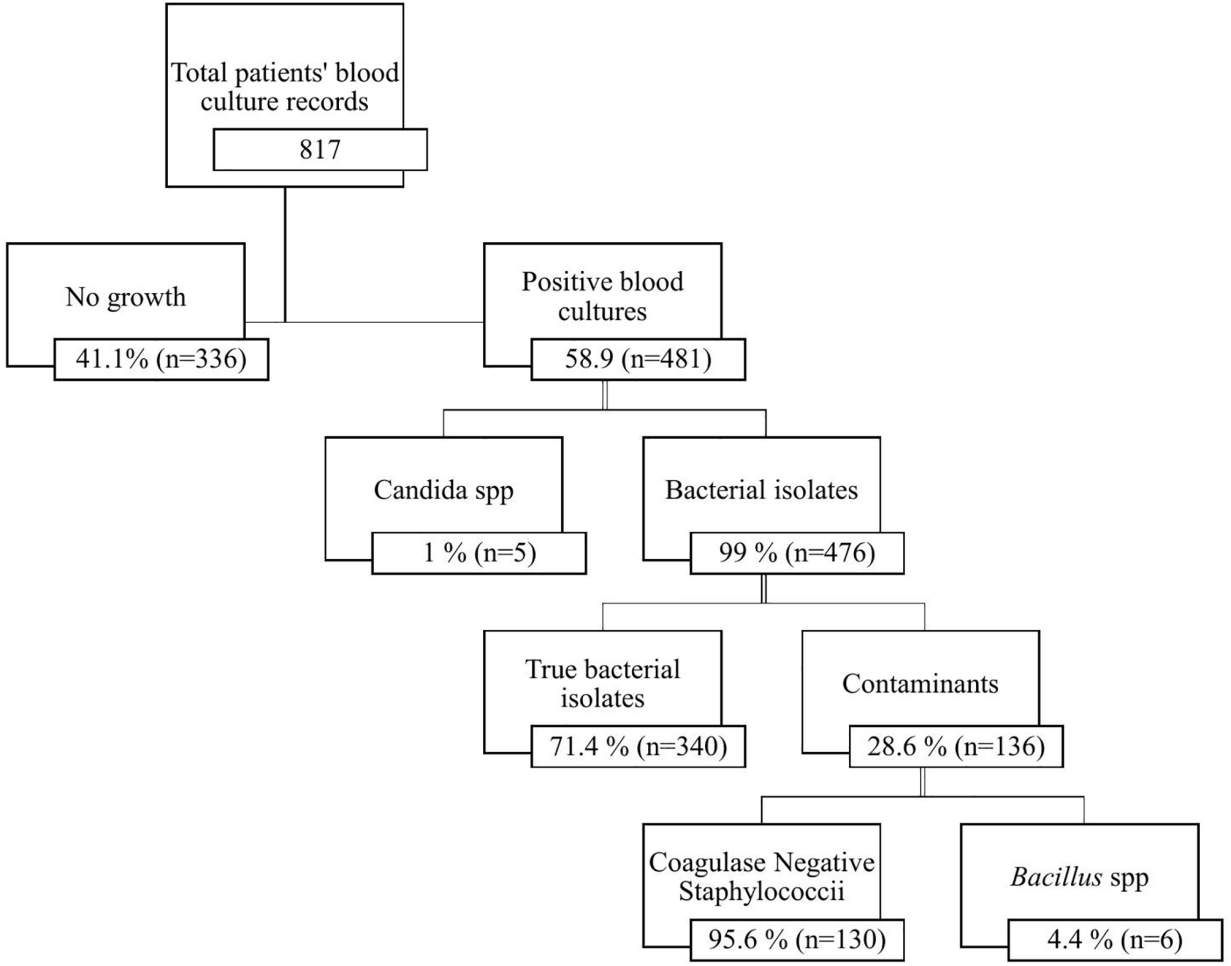
Distribution of positive blood cultures, true bacterial isolates and contaminants at the Amhara Public Health Institute, 2020–2023.

Out of 817 blood culture records retrieved, 58.9% (N = 481) yielded growth, while 41.1% (N = 336) showed no growth. Among the positive cultures, 29.3% (N = 141) were identified as contaminants and excluded from further analysis. These included Coagulase-negative staphylococci (CoNS) (27%, N = 130), *Bacillus* spp. (1.2%, N = 6) and *Candida* spp. (1%, N = 5). The remaining 676 blood culture records were included in the final analysis, of which 50.3% (N = 340) yielded true bacterial pathogens (Fig 1).

**Fig 2:**
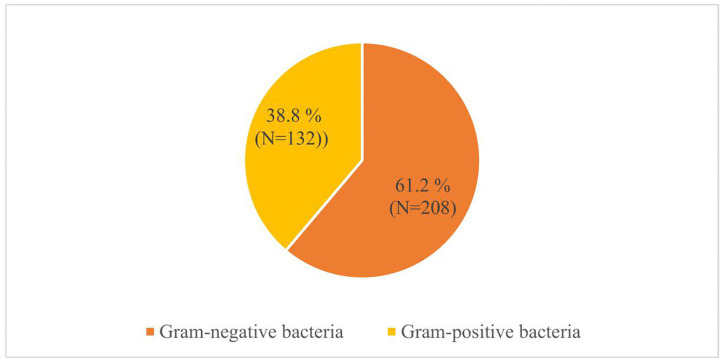
Proportion of Gram-negative and Gram-positive bacterial isolates from patients with suspected bloodstream infections at Amhara Public Health Institute, 2020-2023.

Among the 340 true bacterial pathogens, Gram-negative bacteria were the most frequent isolates at 61.2% (N = 208), while Gram-positive bacteria accounted for the remaining 38.8% (N = 132) (Fig 2).

**Fig 3:**
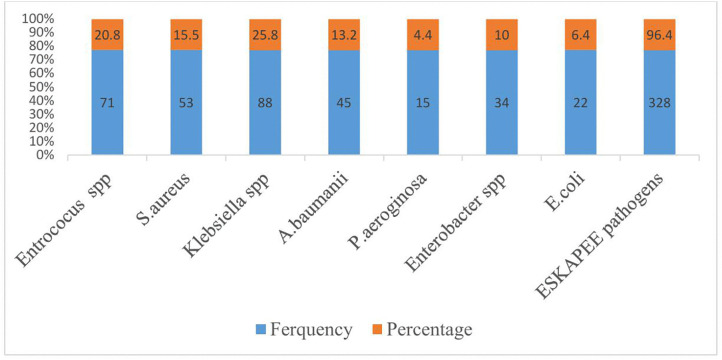
Frequency and distribution of isolated ESKAPEE pathogens from patients with suspected bloodstream infections at Amhara Public Health Institute, 2020-2023.

Among true bloodstream infections, ESKAPEE pathogens accounted for 96.4% (N = 328) of isolates, driven primarily by *Klebsiella* spp. 25.9% (n = 88), *Enterococcus* spp. 20.9% (N = 71), and *S. aureus* 15.6% (N = 53) (Fig 3).

**Fig 4:**
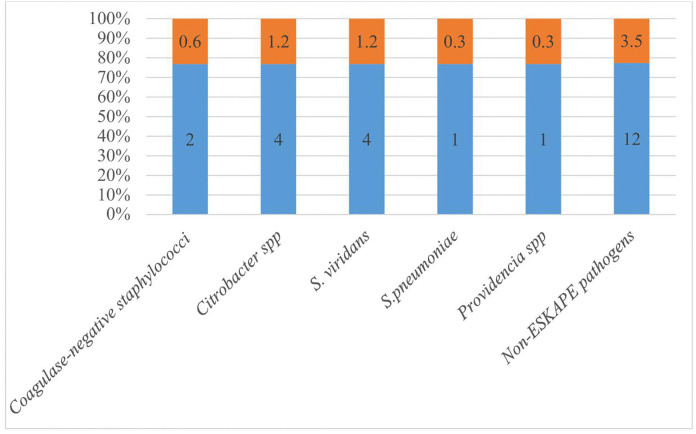
Frequency and distribution of non-ESKAPEE pathogens from patients with suspected bloodstream infections at Amhara Public Health Institute, 2020-2023.

Non-ESKAPEE pathogens were infrequently isolated, accounting for only 3.5% (N = 12) of the true pathogens. Among these, *Citrobacter* spp. and true CoNS represented 1.2% (N = 4) and 0.6% (N = 2) respectively (Fig 4).

### Antimicrobial susceptibility patterns of bacterial isolates

Analysis of the antimicrobial resistance patterns among Gram-positive isolates revealed high resistance rates to oxacillin were 74.5% (N = 41), penicillin was 72% (N = 36), and vancomycin was 51.1% (N = 24). Resistance profiles varied significantly across species. While *Enterococcus* spp. demonstrated high susceptibility to chloramphenicol 77.8% (N = 42), they exhibited substantial resistance to penicillin 71.1% (N = 32) and vancomycin 57.1% (N = 24). Among *S. aureus* isolates, susceptibility to gentamicin was 56.7% (N = 21), but resistance to oxacillin and penicillin reached 75.5% (N = 40) and 80% (N = 4) respectively. In contrast, *S. pneumoniae* and *S. viridans* isolates displayed complete susceptibility to gentamicin, vancomycin, and chloramphenicol. Overall, gentamicin and chloramphenicol demonstrated the highest cumulative efficacy against the Gram-positive pathogens in this study (Table 2).

**Table 2:** Antimicrobial susceptibility pattern of Gram-positive bacteria isolates among patients with suspected bloodstream infections at Amhara Public Health Institute, 2020-2023.

Bacterial Isolates	Patterns	Antimicrobial resistance (%)				
		Chloramphenicol	Oxacillin	Penicillin	Gentamicin	Vancomycin
CoNS	S	NA	1 (50)	NT	1 (50)	NA
	R	NA	1 (50)	NT	1 (50)	NA
*Enterococcus* spp.	S	42 (77.8)	NA	13 (28.9)	NA	18 (42.9)
	R	12 (22.2)	NA	32 (71.1)	NA	24 (57.1)
*S. viridians*	S	NA	NA	NA	NA	4 (100)
	R	NA	NA	NA	NA	0
*S. aureus*	S	NA	13 (24.5)	1 (20)	21 (56.7)	NA
	R	NA	40 (75.5)	4 (80)	16 (43.2)	NA
*S. pneumoniae*	S	NA	NA	NT	NA	1(100)
	R	NA	NA	NT	NA	0
Total	S	42 (77.8)	14 (25.5)	14 (28)	22 (56.4)	23 (48.9)
	R	12 (22.2)	41(74.5)	36 (72)	17 (43.6)	24 (51.1)

**Note:** CoNS, Coagulase-negative staphylococci; NT, not tested; NA, not applicable; spp., species

In this study, Gram-negative bacteria were resistant to commonly used antimicrobials include ampicillin 100% (N = 24), ceftriaxone 92.4% (N = 157), trimethoprim-sulphamethoxazole 89.4% (N = 126), amoxicillin-clavulanic acid 81.1% (N = 103), ceftazidime 80.9% (N = 93), gentamicin 118 76.6% (N = 118), tobramycin 70.3% (N = 109), ciprofloxacin 66.5% (N = 127), and piperacillin-tazobactam 66.7% (N = 14) respectively.

*Klebsiella* species exhibited severe resistance profiles, with *K. oxytoca* reaching 100% resistance to ceftriaxone, ceftazidime, and tobramycin and *K. pneumoniae* demonstrating up to 96.3% (N = 26) resistance to ceftazidime and ceftriaxone 95.5% (N = 63), trimethoprim-sulphamethoxazole 89.6% (N = 60), gentamicin 83.8% (N = 57), amoxicillin-clavulanic acid 80% (N = 48), piperacillin-tazobactam 80% (N = 4), tobramycin 76.1% (N = 35), and ciprofloxacin 69.6% (N = 48) respectively. Chloramphenicol 53.3% (N = 8), imipenem 57.1% (N = 4), and meropenem 87.5% (N = 7) were more effective against *K. pneumoniae*.

**Fig 5:**
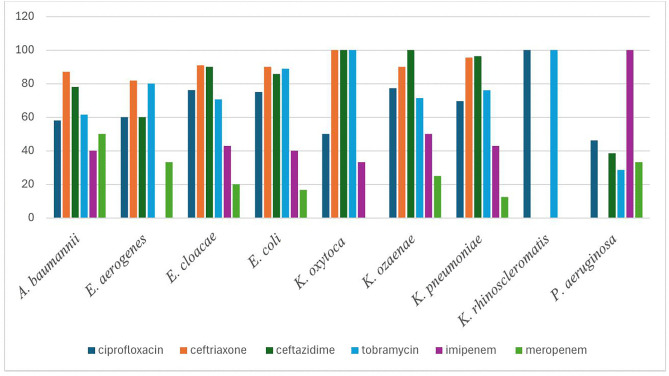
Antimicrobial resistance profiles of Gram-negative bacterial isolates to selected antibiotics.

A. *baumannii* showed high levels of resistance to ceftriaxone 87.1% (N = 27), ceftazidime 78.1% (N = 25), tobramycin 61.5% (N = 24), and ciprofloxacin 58.1% (N = 25). It was found that *A. baumannii* was susceptible to imipenem 60% (N = 6) and meropenem 50% (N = 6). The isolates of *E. coli* showed high resistance to ciprofloxacin 75% (N = 15), ampicillin 100% (N = 12), ceftriaxone 90% (N = 18), tobramycin 88.9% (N = 16), and amoxicillin-clavulanic acid 76.2% (N = 16). Chloramphenicol 50% (N = 4), imipenem 60% (N = 3), and meropenem 83.3% (N = 5) were more effective against *E. coli.* Additional, *E. cloacae* showed high resistance to the following antimicrobials: ampicillin 100% (N = 11), trimethoprim-sulphamethoxazole 95% (N = 19), amoxicillin-clavulanic acid 94.4% (N = 17), ceftriaxone 90.9% (N = 20), ceftazidime 90% (N = 9), chloramphenicol 88.9% (N = 8), gentamicin 77.3% (N = 17), ciprofloxacin 76.2% (N = 16), and tobramycin 70.6% (N = 12). *E. cloacae* were susceptible to meropenem 80% (N = 4) and imipenem 57.1% (N = 4). Notably, *P. aeruginosa* presented a reversal of typical trends, displaying 100% (N = 1) resistance to imipenem but remaining highly sensitive to tobramycin 71.4% (N = 10) and ceftazidime 61.5% (N = 8) resistance (Table 3 and Fig 5).

**Table 3:** Antimicrobial susceptibility pattern of Gram-negative bacterial isolates among patients with suspected bloodstream infections at Amhara Public Health Institute, 2020-2023.

Bacterial isolate	Patterns	Antimicrobial resistance (%)											
		AMP	CAF	CIP	CRO	SXT	GEN	AUG	CAZ	PTZ	TOB	IMP	MER
*A baumannii*	S	NA	NA	18(41.9)	4(12.9)	NA	NT	NA	7(21.9)	3(42.9)	15(38.5)	6(60)	6(50)
	R	NA	NA	25(58.1)	27(87.1)	NA	NT	NA	25(78.1)	4(57.1)	24(61.5)	4(40)	6(50)
*Citrobacter* spp.	S	NA	3(75)	0	0	0	0	1(50)	1(50)	NT	0	0	NT
	R	NA	1(25)	3(100)	3(100)	3(100)	4(100)	1(50)	1(50)	NT	2(100)	1(100)	NT
*E. aerogenes*	S	NA	2(40)	2(40)	2(18.2)	2(18.2)	3(27.3)	2(16.7)	2(40)	0	1(20)	1(100)	2(66.7)
	R	NA	3(60)	3(60)	9(81.8)	9(81.8)	8(72.7)	10(83.3)	3(60)	2(100)	4(80)	0	1(33.3)
*E. cloacae*	S	0	1(11.1)	5(23.8)	2(9.1)	1(5)	5(22.7)	1(5.6)	1(10)	1(50)	5(29.4)	4(57.1)	4(80)
	R	11(100)	8(88.9)	16(76.2)	20(90.9)	19(95)	17(77.3)	17(94.4)	9(90)	1(50)	12(70.6)	3(42.9)	1(20)
*E. coli*	S	0	4(50)	5(25)	2(10)	4(19)	9(45)	5(23.8)	2(14.3)	NT	2(11.1)	3(60)	5(83.3)
	R	12(100)	4(50)	15(75)	18(90)	17(81)	11(55)	16(76.2)	12(85.7)	NT	16(88.9)	2(40)	1(16.7)
*K. oxytoca*	S	NA	1(100)	3(50)	0	0	0	0	0	NT	0	2(66.7)	1(100)
	R	NA	0	3(50)	7(100)	7(100)	7(100)	4(100)	5(100)	NT	5(100)	1(33.3)	0
*K. ozaenae*	S	NA	1(33.3)	2(22.2)	0	1(10)	2(22.2)	3(37.5)	0	NT	2(28.6)	1(50)	3(75)
	R	NA	2(66.7)	7(77.3)	9(90)	9(90)	7(77.8)	5(62.5)	7(100)	NT	5(71.4)	1(50)	1(25)
*K. pneumoniae*	S	NA	8(53.3)	21(30.4)	3(4.5)	7(10.4)	11(16.2)	12(20)	1(3.7)	1(20)	11(23.9)	4(57.1)	7(87.5)
	R	NA	7(46.7)	48(69.6)	63(95.5)	60(89.6)	57(83.8)	48(80)	26(96.3)	4(80)	35(76.1)	3(42.9)	1(12.5)
*K. rhinoscleromatis*	S	NA	0	0	NT	0	0	0	NT	NT	0	1(100)	NT
	R	NA	1(100)	1(100)	NT	1(100)	1(100)	1(100)	NT	NT	1(100)	0	NT
*P. aeruginosa*	S	NA	NA	7(53.8)	NA	NA	6(50)	NA	8(61.5)	2(40)	10(71.4)	0	2(66.7)
	R	NA	NA	6(46.2)	NA	NA	6(50)	NA	5(38.5)	3(60)	4(28.6)	1(100)	1(33.3)
*Providencia* spp.	S	0	0	1(100)	0	0	NT	0	NT	NT	0	NT	1(100)
	R	1(100)	1(100)	0	1(100)	1(100)	NT	1(100)	NT	NT	1(100)	NT	0
Total	S	0	20(43.5)	64(33.5)	13(7.6)	15(10.6)	36(23.4)	24(18.9)	22(19.1)	7(33.3)	46(29.7)	22(57.9)	31(72.1)
	R	24(100)	26(56.5)	127(66.5)	157(92.4)	126(89.4)	118(76.6)	103(81.1)	93(80.9)	14(66.7)	109(70.3)	16(42.1)	12(27.9)

**Note**: AMP, ampicillin; AMC, amoxicillin-clavulanic acid; CAF, chloramphenicol; CRO, ceftriaxone; CAZ, ceftazidime; CIP, ciprofloxacin; GEN, gentamicin; IMP, imipenem; MEM, meropenem; PTZ, piperacilline-tazobactem; SXT, trimethoprim-sulfamethoxazole; NA, not applicable; NT, not tested; spp., species

### Prevalence of MDR, XDR and PDR bacterial isolations

This study analyzed antimicrobial susceptibility test for 50.3% (N = 340) of true pathogenic bacterial isolates. Multidrug-resistant (MDR), extensively drug-resistant (XDR), and pandrug-resistant (PDR) strains were identified; MDR accounted for 43.8% (N = 149), XDR for 30.5% (N = 104), and PDR for 5.3% (N = 18) of the isolates. In ESKAPEE pathogens, MDR, XDR, and PDR were 44.2% (N = 145), 31.4% (N = 103), and 5.1% (N = 17). The non-ESKAPEE pathogens were also 28.6% (N = 4), 7.1% (N = 1), 7.1% (N = 1) MDR, XDR, and PDR, respectively. The following Gram-negative bacteria in particular notable MDR, XDR, and PDR frequencies: *Klebsiella* spp. 81.8% (N = 88), 9.1% (N = 8), 6.8% (N = 6), *Enterobacter* spp.70.6% (n = 24), 14.7% (N = 5), 14.7% (N = 5), *E. coli* 77.3% (N = 17), 13.6% (N = 3) and 9.1% (N = 2) respectively (Table 4).

**Table 4:** Antimicrobial resistance categories (MultiS, MDR, XDR, and PDR) of true bacterial pathogens causing bloodstream infections (N = 340).

Bacterial isolates	Total	MultiS	MDR	XDR	PDR
*Enterococcus* spp.	71	22 (31)	2 (2.8)	47 (66.2)	0
*S. aureus*	53	25 (47.2)	1(1.9)	26 (49)	1 (1.9)
*K. pneumoniae*	70	2 (2.8)	58 (82.8)	6 (8.6)	4 (5.7)
*K.ozaenae*	10	0	7 (70)	2 (20)	1 (10)
*K. oxytoca*	7	0	6 (85.7)	0	1 (14.3)
*K. rhinoscleromatis*	1	0	1 (100)	0	0
*A. baumannii*	45	7 (15.5)	22 (48.9)	13 (28.9)	3 (6.7)
*P. aeruginosa*	15	7 (46.7)	7 (46.7)	1 (6.6)	0
*E. clocae*	22	0	16 (72.7)	2 (9.1)	4 (18.2)
*E. aerogenes*	12	0	8 (66.7)	3 (25)	1 (8.3)
*E. coli*	22	0	17 (77.3)	3 (13.6)	2 (9.1)
ESKAPEE	328	63 (19.2)	145 (44.2)	103 (31.4)	17 (5.2)
*Citrobacter* spp.	4	0	2 (50)	1 (25)	1 (25)
*Providencia* spp.	1	0	1 (100)	0	0
CoNS	2	1 (50)	1 (50)	0	0
*S. pneumoniae*	1	1 (100)	0	0	0
*S. viridans*	4	4 (100)	0	0	0
non-ESKAPEE	12	6 (50)	4 (33.3)	1 (8.3)	1 (8.3)
Total	340	69 (20.3)	149 (43.8)	104 (30.5)	18 (5.3)

**Note**: CoNS, Coagulase-negative staphylococci; spp., species; MultiS, susceptible to all antimicrobial classes; MDR, resistant to at least one agent in three or more antimicrobial categories; XDR, resistant to at least one agent in all but two or fewer antimicrobial categories (i.e., bacterial isolates remain susceptible to only one or two categories); PDR, resistant to all antimicrobial classes [9].

### Factors associated with bloodstream infections

Both bivariable and multivariable logistic regression analyses demonstrated that sex and age were the only independent predictors of a positive culture result, whereas clinical setting and prior antimicrobial use had no statistically significant impact. While males comprised the majority of the study population 58.8% (N = 398), female sex was associated with a significantly lower likelihood of culture positivity (AOR = 0.56; 95% CI: 0.39–0.81; p = 0.002), representing a 44% reduction in odds compared to males. Age-stratified analysis revealed substantially elevated risks of positive cultures in two specific independent predictors relative to elderly patients (≥ 65 years). Specifically, neonates (≤ 28 days) were 4.7 times more likely to test positive (AOR = 4.70; 95% CI: 1.83–12.03; p = 0.001), while young adults aged 15–24 years exhibited the highest risk, with nearly 9.5-fold higher odds of a positive culture (AOR = 9.46; 95% CI: 3.08–29.03; p = 0.001). No statistically significant differences in culture outcomes were observed for the remaining age brackets when compared to the elderly patients (Table 5).

**Table 5:** Bivariable and multivariable logistic regression analysis of factors associated with bloodstream infections among suspected patients at Amhara Public Health Institute, 2020-2023 (N = 676).

Variables	Category		Culture result		AOR (95% CI)	P-value
		Total, N (%)	Positive No (%)	Negative No (%)		
Sex	Female	278 (41.2)	128 (18.9)	150 (22.1)	0.56 (0.39, 0.81)	0.002*
	Male	398 (58.8)	212 (31.3)	186(27.5)	1	
Age group	≤ 28 days	161(23.8)	118 (17.4)	43 (6.3)	4.70 (1.83, 12.03)	0.001*
	29 days – 5 years	188 (27.8)	76 (11.2)	112 (16.5)	1.33 (0.53, 3.35)	0.534
	6 −14 Years	79 (11.6)	34 (5)	45 (6.6)	1.60 (0.59, 4.33)	0.534
	15 - 24 years	57 (8.4)	47 (6.9)	10 (1.4)	9.46 (3.08, 29.03)	0.001*
	25 - 54 years	139 (20.5)	51 (7.5)	88 (13)	1.05 (0.41, 2.71)	0.906
	55 - 64 years	27 (3.9)	5 (0.7)	22(3.2)	0.48 (0.12, 1.88)	0.299
	≥ 65 years	25 (3.6)	9 (1.3)	16(2.3)	1	
Patient’s clinical setting	Inpatient department	602 (89.1)	316 (46.7)	286 (42.3)	0.93 (0.47, 1.72)	0.758
	Outpatient department	74 (10.9)	24 (3.5)	50 (7.3)	1	
Prior antimicrobial use	No	146 (21.6)	54 (7.9)	92 (13.6)	0.66 (0.42,1.03)	0.071
	Yes	530 (78.4)	286 (42.3)	244 (36.1)	1	

**Note**: AOR, Adjusted odds ratio; CI, confidence interval; *statistically significant association

## Discussion

Bloodstream infections (BSIs) remain a primary global driver of severe morbidity and mortality. Because pathogen distribution and antimicrobial resistance trends vary significantly by geographic region, localized surveillance data are vital for optimizing empirical antimicrobial therapy and guiding effective public health strategies.

In the present study, the overall prevalence of true bacterial isolates among patients suspected of bloodstream infections was 50.3% (N = 340). This finding is consistent with previous studies conducted in California (49.5%) [20], China (55.1%, 42.9%) [21,22], and Saudi Arabia (46.6%) [23], Tanzania (45.5%) [24,25], Nigeria (42.8%) [26], Malawi (48.9%) [27], Egypt (50.4%) [28], Ethiopia (56%) [29,30]. These findings reveal a substantial burden of bacterial pathogens within the study population, underscoring a critical regional public health concern that demands targeted intervention. Conversely, the prevalence noted in our study falls below the rates previously documented in other Ethiopian healthcare facility (76.8%) [31]. This variation may be from differences in clinical sample collection protocols, prior empirical antibiotic exposure, or regional variations in patient demographics. Conversely, the positivity rate in our study was higher than the finding from a study conducted in Italy (20.0%) [32], Baghdad (20.8%) [33], Bangladesh (18.8% [34], Maldives (4.9%) [35], Nepal (7.2 % and 7%) [36,37], Tanzania (5.2%) [38], China (7.9%) [39], India (25.3%, 29.8% and 14.2%) [40–42], Asmara (35.5%) [43] and Ethiopia (9.8%, 28.06%, 25.5%) [44–47]. This disparity may be attributed to geographic variations, distinct study periods, and differences in patient demographic profiles, sampling techniques and microbiological methodologies.

In this study, Gram-negative bacteria were identified as the primary cause of bloodstream infections (BSIs), accounting for 61.2% (N = 208) of isolates, while Gram-positive bacteria were responsible for 38.8% (N = 132) of isolates. This clear predominance of Gram-negative pathogens aligns with recent studies conducted in Italy (62.0% vs 37.3%) [48,49], Vietnam (64.12% vs 28.73%, 67.9% vs 31%, 65.7% vs 34.3%) [14,50–52], Nepal (78.7% vs 21.31%, 65% vs 35%) [36,53,54], India 70·0% vs 30%, (56.7% vs 36.7%) [55–57], Saudi Arabia (71.9% vs 28.1%) [58], South Africa (57% vs 36%, 60% vs 33%) [29,18], Zambia (64.8% vs 35.2%) [19], Ethiopia (54.5% vs 45.43%, 60% vs 30.8%, 58% vs 40.4%) [30,31,45]. In contrast to our findings, Gram-positive bacteria are consistently reported as the predominant etiology of bloodstream infections (BSIs) in studies conducted in Bangladesh (68.9% vs 31.1%) [59]), Nepal (64.6% vs 35.4%) [60], China (61.84% vs 38.26%) [61], Ghana (67% vs 33%) [62] and Ethiopia (59.1% vs 40.9%, 77.4% vs 22.6%) [44,47]. This notable predominance may be attributed to the intrinsic resistance characteristic of Gram-negative pathogens, alongside their capacity for prolonged survival and persistence within healthcare environments.

In this study, a substantial proportion of bloodstream infections 96.5% (N = 328), were caused by the emerging ESKAPEE group of pathogens. Among these, *Klebsiella* spp. were the most frequently isolated organisms at 25.8% (N = 88), followed by *Enterococcus* spp. at 20.8% (N = 71), *S. aureus* at 15.5% (N = 53), *A. baumannii* at 13.2% (N = 45), *Enterobacter* spp. at 10% (N = 34), *E. coli* at 6.4% (N = 22) and *P. aeruginosa* at 4.4% (N = 15) respectively. This pathogen distribution aligns closely with studies done from Perugia (78.9%) [63], Romania (75.6%, 97%) [64,65], Italy (38.7%) [66], India (77.6%) [67], Turkey (73.2%) [68], China (73.1%) [69], Zambia (25.7%) [70] and Ethiopia (67%, 65.3%) [31,71]. This pattern is largely due to the widespread resistance of these pathogens to common first-line antibiotics, creating significant therapeutic challenges in clinical management.

In our study, resistance among Gram-positive pathogens was critically high for oxacillin 74.6% (N = 41), penicillin 72% (N = 36), and vancomycin 51.1% (N = 24). Similar escalating resistance profiles have been documented in blood culture studies from other parts of the countries, Turkey (penicillin 93.8%, oxacillin 44.3%) [72], Bangladesh (penicillin 92%) [59], India (oxacillin 40.6% and vancomycin 11.9%, oxacillin 70.6% and vancomycin 21.6%) [73,74], Saudi Arabia (oxacillin 12.3%) [75], Macedonia (penicillin 100% and vancomycin 33,3%) [76], Asmara (oxacillin 80.4%) [43], Ethiopia (penicillin 60%, oxacillin 66.7%) [77], (oxacillin 63%), (oxacillin 62.5% and penicillin 81%), (penicillin 92.4%), (penicillin 85%, oxacillin 38% and vancomycin 94%) [46,78–82] have also reported comparable finding on this regards. This may be due to several mechanisms, including enzymatic degradation of antimicrobials, alterations in drug targets, reduced drug uptake, and the formation of biofilms. While some studies in Colombia report lower resistance rates to oxacillin (40.8%) and penicillin (51%) [83]. This variation may be attributed to differences in study population, variations in diagnostic methodologies, and the continuous evolution of antimicrobial resistance profiles over time.

The present study indicates a high level of antimicrobial resistance in *Enterococcus* spp. to penicillin 71.1% (N = 32) and vancomycin 57.1% (N = 24). This alarming resistance profile aligns with reports from China, where *Enterococcus* spp. demonstrated a penicillin resistance rate of 94.1% and a vancomycin resistance rate of 31.1% [84,85]. These high levels of antimicrobial resistance may be driven by several factors, including intrinsic resistance, acquired resistance mechanisms, and the selective pressure of antimicrobial use.

In the present study, *S. aureus* isolates demonstrated substantial resistance to penicillin 80% (N = 4) and oxacillin 75.5% (N = 40). These findings are consistent with surveillance data from Turkey, where oxacillin resistance rates among *S. aureus* isolates ranged from 50.8% to 66.0% [86,87], Italy (oxacillin 36.4%) [49], Iran (penicillin 91.8%) [88], Vietnam (oxacillin 77.2%), Bangladesh (penicillin 87.5%) [89], Saudi Arabia (penicillin 98%) [75], Nigeria (37.5%) [90], Asmera (oxacillin 80.4%) [43], Ethiopia penicillin resistance rates ranging from 83.5% to 86.7%, and oxacillin resistance from 24.5% to 88.9% [8,31,46,79,80,91–93]. This is might be due to the presence of the mecA gene, which encodes for the altered penicillin-binding protein PBP2a, leading to resistance to beta-lactam antimicrobials like penicillin and oxacillin. The current study’s findings may deviate from Bangladesh oxacillin (7.40%) [89] and Ethiopia (penicillin 29.4%) [94].This might be due to variations in bacterial strains, genetic makeup, and the presence of other resistance mechanisms beyond the standard mecA-mediated resistance.

The Gram-negative bacterial isolates exhibit a high level of resistance to ampicillin 100% (N = 24), ceftriaxone 92.4% (n = 157), trimethoprim-sulphamethoxazole 89.4% (n = 126), amoxicillin-clavulanic acid 81.1% (N = 103), ceftazidime 80.9% (N = 93), gentamicin 118 76.6% (N = 118), tobramycin 70.3% (N = 109), piperacillin-tazobactam 66.7% (N = 14) and ciprofloxacin 66.5% (N = 127) respectively. These resistance profiles closely align with the findings reported in Palestinian resistance to ampicillin (87.2%) [95]. Similarly, multiple studies from Ethiopia have documented comparable trends, with ampicillin resistance ranging from 66.0% to 93.0%, amoxicillin-clavulanic acid from 83.9% to 88.7%, cotrimoxazole from 64.1% to 75.5%, and ceftriaxone at 82.6% [8,77,80,82,96–99] respectively. This pervasive increase in antimicrobial resistance is likely driven by inherent bacterial defense mechanisms, the horizontal acquisition of resistance genes, and the selective pressure exerted by widespread antimicrobial utilization. Conversely, resistance rates in the present study were notably higher than those reported in Nepal, where gentamicin and ampicillin resistance were limited to 7.4% and 66.6% [100] respectively. These discrepancies are most likely attributable to regional variations in antibiotic prescribing behaviors, local healthcare infrastructure, and the distinct geographical distribution of resistant bacterial strains.

In the present study, *K. pneumoniae* isolates demonstrated high resistance rates to ceftazidime 96.3% (N = 26), ceftriaxone 95.5% (N = 63), trimethoprim-sulphamethoxazole 89.6% (N = 60), gentamicin 83.8% (N = 57), amoxicillin-clavulanic acid 80% (N = 48), piperacillin-tazobactam 80% (N = 4), tobramycin 76.1% (N = 35), and ciprofloxacin 69.6% (N = 48) respectively. Similar high resistance profiles have been documented across sub-Saharan Africa. For instance, studies in Zambia reported substantial resistance to gentamicin (81%) and fluoroquinolones (80.8%) [101]. In Cameroon, notable resistance was observed against beta-lactams (72%), phenolics (62.30%), and quinolones (60.41%) [102]. Furthermore, data from Ethiopia highlighted significant resistance to ampicillin (75%), amoxicillin-clavulanic acid (62.5%), ceftriaxone (62.5%), and trimethoprim-sulfamethoxazole (50%) [94]. The elevated resistance rates observed in *K. pneumoniae* are typically driven by inherent bacterial defense mechanisms, selective pressure from widespread antimicrobial misuse, and the horizontal transfer of resistance genes between bacterial populations. Conversely, certain strains demonstrated continued susceptibility to specific reserve antibiotics. In the present study, *K. pneumoniae* displayed the highest susceptibility to meropenem 87.5% (N = 7), followed by imipenem at 57.1% (N = 4) and chloramphenicol at 53.3% (N = 8). These susceptibility trends align closely with previous findings from Cameroon, which similarly recorded high susceptibility rates for imipenem (91.7%), co-trimoxazole (78.1%), tobramycin (71.8%), and gentamicin (56.2%) [102,103].

In this study, *A. baumannii* isolates demonstrated notable resistance to several common antimicrobials, including ceftriaxone 87.1% (N = 27), ceftazidime 78.1% (N = 25), tobramycin 61.5% (N = 24) and ciprofloxacin 58.1% (N = 25). These resistance profiles are comparable with findings reported in previous studies in Greece (80% to 100%) [104], though notably higher than those observed in Nepal 26.8% [42]. Conversely, the isolates in this cohort retained moderate sensitivity to carbapenem reserve antibiotics, specifically imipenem at 60% (N = 6) and meropenem at 50% (N = 6). Generally, *A. baumannii* develops resistance to these beta-lactam agents through four primary mechanisms: enzymatic inactivation by hydrolysis, increased drug efflux, decreased membrane influx, or structural protection of the antimicrobial target site.

In the present study, the overall prevalence of multidrug-resistant (MDR) isolates was 43.6% (N = 149). This rate is slightly lower than previous findings reported in West Africa (59%), and various regions in Ethiopia (47.1% to 61.6%) [130–133]. Considerably higher MDR resistance rates have been documented elsewhere, such as in Bangladesh (83.4%) [59], Zambia (90%) [87], and in other Ethiopian studies where rates ranged from 68.2% to 84.4% [44,57,95,97,99,134]. Conversely, markedly lower MDR prevalence were reported in New Zealand (3.4%) [135], Italy (6%) [136], South Korea (24.7%) [137], and a specific localized study in Ethiopia (19.7%) [138].

In the present study, the overall prevalence of multidrug-resistant (MDR) isolates was 43.6% (N = 149). This rate is slightly lower than previous findings reported in West Africa (59%) [105], Nigeria (57.4%) [106], and various regions across Ethiopia (47.1% to 61.6%) [107–110] . However, more elevated resistance rates have been reported in Bangladesh (83.4%) [59], Zambia (90%) [87], and other studies in Ethiopia (ranging from 68.2% to 84.4%) [44,57,95,97,99]. Conversely, lower MDR rates were documented in New Zealand (3.4%) [111], Italy (6%) [112], South Korea (24.7%) [113], and a specific study in Ethiopia (19.7%) [114]. Among the ESKAPEE pathogens, 44.2% (N = 145) of the isolates exhibited multidrug resistance. This aligns with previous regional and international data showing high resistance profiles, such as in Greece (*P. aeruginosa* at 30% and *A. baumannii* at 97%)[115], Turkey (50.86%) [15], and Ethiopia (*A. baumannii* at 100%, MRSA at 90%, and *K. pneumoniae* at 67%) [44,88]. Because the highest rates of MDR were predominantly observed among Gram-negative bacterial isolates, conducting routine antimicrobial susceptibility testing is essential to guide effective treatment and manage the spread of bacterial infections. In the present study, the prevalence rates of extensive drug-resistant (XDR) and pandrug-resistant (PDR) bacterial isolates were 30.4% (N = 104) and 5.3% (N = 18) of bacterial isolated. These findings are consistent with those reported from Greece (22%) [104], Nigeria XDR (11.9%) and PDR (4.0%) [116], Addis Ababa XDR (22.4%) and (4%) [117] respectively. Our study finding were higher than others studies, in Canada XDR (2.6%) [118], Saudi Arabia (3.5%) and no PDR were isolated [119], India (5.4%) [120]. However lower than studies conducted in Iran 96.4% were XDR and 76.4% were PDR [121], Pakistan XDR (51%), (54.7%) [122,123], Saudi Arabia XDR (10%) and PDR (6%), [124], Addis Ababa XDR, and PDR were 32.2% and 7.3% [45], Gondar XDR (24.8%) and PDR (4.8%) [125], Bahir Dar XDR, and PDR were 53%, 43% and 2% [71] respectively. This may be due to increased selection pressure caused by self-medication, misuse and overuse of antimicrobials and absence of an appropriate antibiotic policy.

In the present study, both bivariable and multivariable logistic regression analyses revealed that female sex was associated with a significantly lower likelihood of culture positivity (AOR = 0.56; 95% CI: 0.39–0.81; *p = 0.002*), representing a 44% reduction in odds compared to males. This finding is strongly supported by previous studies in Norway that females had lower risk of BSI compared to males (AOR) = 1.4, 95% CI = 1.32–1.59) [126], Ireland (AOR)=1.3, 95% CI = 1.31–1.46) [127], Maldives (59%) [35], Brazil (AOR) = 2.9, 95% CI = 1.18–7.47, P = 0.02) [128] and Minnesota BSIs were less common in females than males (23.8% vs 13.9%; P = 002) [129]. This might be due to differences in biological immunity, anatomical differences and behavioral factors. While others studies in Tanzania indicated that female (AOR) =2.2, 95% CI = 1.01, 4.80; p = 0.048) were associated with increased odds of BSI [130]. In contrary a study in Iran reported that no significant association was found between BSIs and gender (P = 0.70) [131].

According to our data, neonates *(*≤ 28 days*)* were 4.7 times more likely to test positive (AOR = 4.70; 95% CI: 1.83–12.03; p = 0.001), while young adults aged 15–24 years exhibited the highest risk, with nearly 9.4-fold higher odds of a positive culture (AOR = 9.46; 95% CI: 3.08–29.03; p = 0.001). This finding is consistent with other studies reported in Malaysia significantly associated with 28-day with increased BSI (AOR) =1.0: 95% CI = 1.03–1.09; p < 0.001) [132], South Korea (AOR) =2.1, 95% Cl = 1.18–3.77, P = 0.005) [133], Sweden (AOR) = 5.5, 95% CI = 2.8–11.0) [134], Ethiopia (AOR) = 3.4, 95% CI  =  1.63–7.11, p = 0.001) [135]. This result was different from studies conducted China [136] showed that older adults are more prone to bloodstream infection due to aging organ function, low immunity and underlying disease.

This study has several limitations. Its retrospective design introduces inherent data constraints, and resource limitations precluded anaerobic blood cultures, potentially lowering the overall diagnostic yield. Additionally, antimicrobial susceptibility testing was restricted to disk diffusion without determining minimum inhibitory concentration (MIC) values. Lastly, because molecular characterization was not performed, specific resistance genes could not be identified.

## Conclusion and recommendations

This study highlights a critical public health crisis with a 50.3% bacterial bloodstream infection rate, dominated by Gram-negative and ESKAPEE pathogens. Resistance is alarmingly high, with over 43% multidrug-resistant strains and extensive failure of first-line drugs like ampicillin and ceftriaxone, particularly risking males, neonates, and young adults. To combat this, healthcare facilities must immediately restrict antibiotic misuse through strict stewardship, update empirical guidelines, and upgrade routine lab testing. Additionally, future research must implement anaerobic culturing, quantitative MIC testing, and molecular sequencing to accurately track and manage these drug-resistant strains.

## Supporting information

S1 File Excel raw data. (XLSX)

## Abbreviations

AOR: adjusted odds ratio
APHI: Amhara public health institute
BSI: blood stream infection
CLSI: clinical laboratory standards institute
CoNS: coagulase-negative staphylococci
ESKAPEE: *Enterococcus* spp., *S. aureus*, *K. Pneumoniae*, *A. baumannii*, *P. aeruginosa*, *Enterobacter* spp. and *E. coli*
MRSA: methicillin resistant *S. aureus*
