# Supplementary material for: Bacterial profile and antimicrobial resistance patterns of bloodstream infections at Amhara Public Health Institute, Bahir Dar, Ethiopia
Source: PLoS One. 2026 Jul 27;21(7):e0354710. doi: 10.1371/journal.pone.0354710 (PMC13405292; doi:10.1371/journal.pone.0354710)
Supplement: Attachment — Submitted filename: Review file.pdf [file pone.0354710.s002.pdf]

**Corrections required:**

In general:

1. Scientific names of organisms are not in italics.
2. The “spp.” need not be in italics.
3. Digits after decimal point should be uniform.
4.

In abstract:

1. It is mentioned that “..all aged groups..” were included. However, the main article shows not only aged people, but a wide range of patients were included in the study.
2. Why was Gram staining of blood done? It is redundant to diagnose infection.
3. Only 24 hours of incubation of the specimen is mentioned which is inadequate for blood culture.

In main article:

1. Line 69: Correction of “E.colli” is required.
2. Line 71: It should be BSI, not BIS.
3. Line 129: As per Oxoid website, their product is tryptone soya broth, not tryptic soya broth. Confirmation of the product used is recommended.
4. Line 133: Why was the range of 35 to 37$^{o}$C kept for incubation? Was there any reason for changing the temperature during actual work? Please specify.
5. Line 139: Only MacConkey is mentioned. Whether agar or broth is missing.
6. Lines 149-151: Identification of *Enterococcus* is missing.
7. Line 154: Why was normal saline used to incubation instead of broth? Any references for this method of AST?
8. Line 159: Spelling is Mueller-Hinton agar, not Muller-Hinton agar.
9. Line 201: ATCC should not be within brackets.
10. Line 205-208: The sentence is unclear.
11. Lines 310-312: Resistance to number of antibiotics is specified in table as R1, R2, etc. It does not fit the MDR criteria.
