# Supplementary material for: Bacterial profile and antimicrobial resistance patterns of bloodstream infections at Amhara Public Health Institute, Bahir Dar, Ethiopia
Source: PLoS One. 2026 Jul 27;21(7):e0354710. doi: 10.1371/journal.pone.0354710 (PMC13405292; doi:10.1371/journal.pone.0354710)
Supplement: Attachment — Submitted filename: Response to Reviewers.docx [file pone.0354710.s004.docx]

Dear Dr. Mengistu,

PhD Academic Editor
PLOS ONE

Authors' response to reviewers' comments:

Reviewer #2: All the comments were addressed well. The declaration section needs improvement during/or before proofreading, especially the author's contribution should be similar to the submission systems.

**Replay:** Dear reviewer, many thanks for your remark. The declaration section, which primarily addresses the author's contribution, has been updated in response to your comments.

Reviewer #3: Dear Editor
This manuscript is about " Bacterial profile and antimicrobial resistance patterns of blood culture isolates at Amhara Public Health Institute, Bahirdar, Ethiopia: Four-year retrospective study". It is a good manuscript, but it is not suitable for publishing in PLOS ONE. This study is just a simple report of bacterial blood culture and the frequency of their antibiotic resistant.

**Replay:** Dear reviewer, I really value your comments. Bloodstream infection-related antimicrobial resistance is a global concern, yet, managing blood stream infection in Ethiopia is hampered by lack of bacteriological support needed for antimicrobial stewardship, and background data needed for empirical treatment. In addition, diagnostic culture laboratories for evaluating antimicrobial resistance are intermittent due to resources constraints, poor microbiology laboratory infrastructure, and a shortage of trained personnel. To help with life-saving clinical decisions, the clinical Laboratory Standards Institute (CLSI) recommended antimicrobials selected depending on bacterial profile, antimicrobial resistance patterns and the site of infection. To help clinicians find alternative treatments for managing bloodstream infections, this study will give them up-to-date information. It will also help other relevant entities evaluate and implement antibiotic resistance caused by bloodstream infection prevention and control strategies.

Reviewer #4: This manuscript narrates the Bacterial profile and antimicrobial resistance patterns of blood culture isolates. The study design is thorough and contextualizes their findings within the urgent national issue of antibiotic resistance in connection with BSI. At the same time, the work repeats a familiar approach without advancing new methodologies for the identification of bacteria and drug resistance. Speciation of many bacteria and Candida is not done. Besides, BSI-causing anaerobic bacteria are not included. The English part of the manuscript needs to be revised.

**Replay:** Dear reviewer, thank you so much for your comment. This is a retrospective study that provides updated information to the concerned body and encourages the scientific community to carry out further research in this area. Blood culture isolates, including both bacterial and fungal isolates, were identified by conventional methods to genus and species level. However, to provide comprehensive information on resistance patterns, lump them together.

Recommendations
The title should be modified
Bacterial profile and antimicrobial resistance patterns of bloodstream infections at Amhara Public Health Institute, Bahirdar, Ethiopia: Four-year retrospective study

**Replay:** Dear reviewer, thank you so much for your comment. We have corrected the title based on your suggestion.
Abstract
1. The abstract should be improved

**Replay:** Dear reviewer, thank you so much for your comment. We have properly revised the abstract section.
2. Replace specimen with samples throughout the manuscript

**Replay:** Dear reviewer, we corrected based on your suggestions. However, a sample is one or more parts taken from a specimen. However, a specimen is a discrete portion of blood taken from the human body for examination, analysis, or study (ISO 15189 Fourth edition 2022)
3. Line 34 The way the overall culture's positive findings are presented in the results is not logical.

**Replay:** Dear reviewer, thank you very much for your feedback. It has been fixed.

4. Standard bacteriological techniques were followed during blood collection. What does it mean? Have authors followed standardized manual blood culture methods following WHO recommendations for resource-limited settings?

**Replay:** Dear reviewer, I appreciate your kind suggestions very much. I am referring to using WHO-recommended standardized manual blood culture techniques in areas with limited resources.

5. Techniques used for the AST test have to be mentioned

**Replay:** We appreciate your kind words. We included it.
6. Is this a work intended to show the problems of ESKAPE pathogens in BSI

**Replay:** Dear reviewer, thanks for your input. This study highlights that the most predominant bacteria isolated in BSIs are the ESKAPE bacteria, which are a major concern due to their multidrug resistance (MDR) profiles and their relevance in nosocomial settings. The Infectious Diseases Society of America coined the acronym ESKAPE to emphasize these pathogens’ ability to escape or evade common therapies through antimicrobial resistance (1).

7. How about Community-onset BSIs

**Replay:** Thank you for your kind inquiries. Instead of community-onset BSIs, this study predicts hospital-acquired infections, BSIs, and other illnesses based on a patient's clinical history.
8. What is the criteria for including patients for Nosocomial BSI with ESKAPE pathogens

**Replay:** Dear reviewer, thank you for your kind remarks. Based on symptoms and clinical presentation, Nosocomial BSI is considered; the infection cannot be present at admission; rather, it must develop at least 48 hours after admission.

9. The acronym has now been revised as ESKAPEE. So update the results

**Replay:** We are grateful for your warm remarks. We have included and updated the results. ESKAPE is an acronym comprising the scientific names of six highly virulent and antibiotic-resistant bacterial pathogens, including: *Enterococcus* spp, *Staphylococcus aureus*, *Klebsiella pneumoniae*, *Acinetobacter baumannii*, *Pseudomonas aeruginosa*, and *Enterobacter* spp. The acronym is sometimes extended to ESKAPEE to include *E.coli*.

10. Why didn’t the authors include the WHO priority pathogens in their study?

**Replay:** Dear reviewer, twenty-four pathogens, representing fifteen families of antibiotic-resistant bacterial pathogens, are listed as WHO priority pathogens for 2024. *Acinetobacter bumannaii, Pseudomonas aeruginosa, Klebsiella pneumonia, E. Coli, Enterococci, Staphylococcus aureus*, and other notable bacteria were categorized as important and high-priority bacterial isolates (2). So, our study included WHO priority pathogens.

11. Results of the overall antibiogram profile have to be included, rather than focusing only on ESKAPE pathogens. Results of drug resistance have to be revised by including the names of antibiotics versus the percentage of resistance

**Replay:** Dear reviewer, we appreciate your insightful remarks. We included an overall antibiogram profile of both Gram-positive and Gram-negative bacterial isolates.
12. Avoid repetitive citation of the abbreviation on lines 24 and 47

**Replay:** Dear reviewer, We have made changes.
13. Use the microbiological rule of abbreviating bacteria. Lines 39 and 40

**Replay:** Dear reviewer, we appreciate your kind words. We have fixed it.

14. Parentheses have to be standardized. N should be placed inside the bracket

**Replay:** Dear reviewer, we appreciate your kind words. We have corrected it.
15. If P values are reported, please follow standard conventions for decimal places: for P values less than .001, report "P<.001"; for P values between .001 and .01, report the value to the nearest thousandth; for P values greater than or equal to .01, report the value to the nearest hundredth; and for P values greater than .99, report as "P>.99."

**Replay:** Dear reviewer, we appreciate your kind words. We have corrected it.
16. In conclusion, the authors mentioned the carbapenem resistance without indicating any findings in the results session

**Replay**: Dear reviewer, we value your opinions. We have corrected it.
17. What about recommending an antimicrobial stewardship programe?

**Replay:** Dear reviewer, Hospitals and other healthcare facilities in the Amhara region have an antimicrobial stewardship program, however, it is not fully functional and not consist of core members like microbiologists. This limits the ability to detect antibiotic resistance in BSI infections and select appropriate treatment management alternatives.

18. Is there any existing antimicrobial stewardship programe in the study setting?

**Replay:** Dear reviewer, the Amhara Public Health Institute does not have an AMS program, but it does serve as a coordinating regional center for AMS and AMR-related programs.

Introduction
It is too general
Clearly state the problem, causes, and outcomes of BSI
Include details of BSI-associated morbidity in Ethiopia
What type of Research gap exists in the study area?
Include some lines on how BSI infection is treated/managed in Ethiopia

**Replay:** Dear reviewer, thank you so much for your comment. We have revised introduction part based on given comments.
Methods
Study area: Remove irrelevant information for international readers from lines 102 to 109.
Should include cases and fatality related to BSI in terms of morbimortality has to be introduced in the study setting. Standard treatment guidelines of BSI in Ethiopia
Bloodstream infections should be detected through standardized manual blood culture methods following WHO recommendations for resource-limited settings. There is no reference in this regard. Factors that may influence the recovery of pathogens from the blood include the timing of blood collection, the number of sets collected, and blood volume.
**Replay:** Dear reviewer, thank you so much for your comment. We have revised methods
 part based on given comments.
True BSIs should be distinguished from potential contaminants (e.g., Micrococcus spp., Bacillus spp. Other than B. anthracis, or coagulase-negative staphylococci (CoNS) using standardized definitions adapted from the CDC guidelines. However, this is missing in this study.
**Replay:** Dear reviewer, thank you so much for your comment. We have a standard operating procedure for manual blood culture methods to rule out which were true priority pathogen or potential contaminants. For common skin contaminants, the number of positive cultures may be used as a means of differentiating contamination from bacteremia. However, most of the published literature in this regard has focused on hospital-onset coagulase-negative staphylococcal infections. For this reason, we have not perform AST for all isolated organisms including CONS (N=128) and Bacillus spp (N=6) considered as contaminates. Additionally, included operational definitions for both true priority pathogen and potential contaminants.

Why this study excludes Community-onset BSIs ?

**Replay:** Dear reviewer, thank you so much for your comment. We did not excluded patients with community-onset BSIs, fungal BSI and all patients with hospitalization <48 hours. In this study patients clinical history predicts to BSIs, hospital-acquired and other infections rather than Community-onset BSIs .The inclusion criteria were suspicion of BSIs; complete records including demographic variables, clinical setting, clinical history, previous antimicrobial use, year of diagnosis, blood culture identification and AST results and presentation at any of the following inpatient or outpatient units.
How only ESKAPE pathogens associated BSI is highlighted?

**Replay:** Dear reviewer, thank you so much for your comment. ESKAPE pathogens are a major cause of bloodstream infections (BSI), accounting for a significant proportion of cases, with our studies reporting prevalence rates of 64.3 % (N=306) of all BSI isolates in patients with suspicion of BSIs. Their ability to "escape" the effects of common antibiotics highlight a serious global health threat, leading to higher healthcare costs, longer hospital stays, and increased mortality compared to non-ESKAPE infections (3) .

What is the overall prevalence of ESKAPE pathogens in the study settings

**Replay:** Dear reviewer, in this study overall prevalence ESKAPE pathogens were 64.3 % (N=306)**.**

Is there any data related to the upsurge of ESKAPE pathogens in the hospital?

**Replay:** Dear reviewers, thank you so much for your comment. There are evidence support the upsurage of ESKAPE pathogens in hospitals in Ethiopia, Felege Hiwot Comprehensive Specialized Hospital 67%(N=1667), Arba Minch General Hospital 65.3% (N=207) and hospitals in Romania 97% (N=4164) of the isolates.

Is there any local antibiotic policy used in the hospital to manage ESKAPE pathogens?
**Replay:** Dear reviewer, thank you so much for your comment. There were compressive local antibiotics polices in health facilities /hospitals to curb the spread of ESKAPE pathogens. However antibiotic policy in a health-care setting couldn’t not efficient due to a number of factors, lack commitment of top management, absence of multidisciplinary antibiotic management team with 6–10 members with expertise and experience in different subjects (usually infectious diseases, internal medicine, surgery, pediatrics, clinical microbiology, pharmacology and hospital pharmacy), skills gap to conduct literature and systematic reviews and lack of inputs from all stakeholders in order to ensure “ownership” of guidelines.

In the results, authors mentioned Candida. However, methodology for its isolation and identification are missing.

**Replay:** Dear reviewer, thank you so much for your comment. Candidemia is a leading cause of healthcare-associated BSI, with all-cause in-hospital mortality reaching 30% in the United States (Toda M et al, 2019). We have included isolation and identification techniques.

What is the selection criterion for antibiotics?

**Replay:** Dear reviewer, thank you so much for your comment. Antibiotics were selected based on specimen type and types of infection using CLSI 2024 M 100 ED 34.

Accurate identification of bacterial species and susceptibility testing is important, but I am not convinced that they have accurately identified the species level of the isolates in their study.
**Replay:** Dear reviewer, thank you so much for raising your concerns. However blood specimen collection, identification, and antimicrobial susceptibility testing for this study were conducted at Amhara Public Health Institute of Bacteriology Reference Laboratory, which was accredited with full scope for a consecutive four years by (ENAO).
Staphylococcus (CONS), Proteus and Klebsiella are heterogeneous group of pathogenic organisms. It is not acceptable to lump them together, considering the objectives of the study.
**Replay:** Dear reviewer, I appreciate your comment. I didn't group them together. But Proteus species did not isolate in our study.

The authors are advised to classify the tested isolates to MDR , XDR, and PDR as described by Magiorakos et al.

**Replay:** Dear reviewer, considering your input, we have included it.

Why authors didn’t inspected MRSA, since these are the WHO prioritized drug resistant bacteria, since the prevalence of these bacteria are frequently appearing in the papers from Ethiopia.

**Replay:** Dear reviewer, I greatly appreciate your comment. We reported that the Cefoxitin disc (30 μg), a serogate marker for oxacillin, was evaluated against *Staphylococcus aureus*. It is reported that if oxacillin is resistant, it is also resistant to methicillin.
Why authors didn’t check the presence of extensive drug resistance (XDR) and pan-drug resistance (PDR), even though the MDR is 68%.

**Replay:** Dear reviewer, considering your input, we have included it.
Details of the vital signs of patients have to be included?

**Replay:** Dear reviewer, to inform you that this studies information was generated from blood culture test orders for initial treatment management of patients. Secondly analyses conducted for research purpose. We could not find details vital signs of patients in the bacteriology reference laboratory during data collection.
Predetermined sample size calculation is missing

**Replay:** Dear reviewer, in retrospective studies, sample size calculation can be somewhat challenging but is not impossible; it is due to the nature of the data being analyzed and typically collected for other purposes. However, we have included records of all BSI suspected patients that meet the inclusion criteria.
Precisely include the eligibility criteria

**Replay:** Dear reviewer, considering your input, we have included it.
The details statistical analysis done are vague. Include all variables regardless of their p-value in the univariate analysis to avoid missing important confounders.

**Replay**: Dear reviewer, considering your input, we have included it.
Strengthen the discussion. Deepen the critical analysis by comparing findings with other recent plant-based antimicrobial studies (especially >2020).

**Replay:** Dear reviewer, considering your input, we have included it.
Discuss limitations more transparently

**Replay:** Dear reviewer, considering your input, we have included it.

1.	Rice LB. Federal funding for the study of antimicrobial resistance in nosocomial pathogens: no ESKAPE. The University of Chicago Press; 2008. p. 1079-81.

2.	Sati H, Carrara E, Savoldi A, Hansen P, Garlasco J, Campagnaro E, et al. The WHO Bacterial Priority Pathogens List 2024: a prioritisation study to guide research, development, and public health strategies against antimicrobial resistance. The Lancet infectious diseases. 2025.

3.	Marturano JE, Lowery TJ, editors. ESKAPE pathogens in bloodstream infections are associated with higher cost and mortality but can be predicted using diagnoses upon admission. Open forum infectious diseases; 2019: Oxford University Press US.
