# Supplementary material for: Bacterial profile and antimicrobial resistance patterns of bloodstream infections at Amhara Public Health Institute, Bahir Dar, Ethiopia
Source: PLoS One. 2026 Jul 27;21(7):e0354710. doi: 10.1371/journal.pone.0354710 (PMC13405292; doi:10.1371/journal.pone.0354710)
Supplement: Attachment — Submitted filename: Response_to_Reviewers_auresp_2.docx [file pone.0354710.s005.docx]

Dear Dr. Mengistu,

PhD Academic Editor
PLOS ONE

Authors' response to reviewers' comments:

**Reviewer #2:**

1. The disk diffusion method (Kirby–Bauer) is useful for routine AST, but it has limitations when classifying advanced resistance categories like XDR and PDR:

**Response:** Thank you for taking the time to review our work and for raising such thoughtful and critical questions. To address these limitations, we have strict adherence to standard testing protocol, Standardized composition of the medium (MHA), Specimen-species-specific antimicrobial susceptibility testing and standardized antibiotic discs used to minimize the influence of variables. Additionally, we utilized recent CLSI guidelines for updating breakpoints and interpretation to improve the accuracy and reliability of AST results.

1.1. XDR and PDR definitions require testing against all or nearly all relevant antimicrobial categories.

**Response:** Dear reviewer, thank you so much for your comment. Multiple studies and standard protocols (CLSI) indicate that disc diffusion (Kirby-Bauer testing) for MDR/XDR/PDR determination typically involve 7–12 distinct antibiotics representing multiple classes: so that, we have tested 12 antibiotics for MDR/XDR/PDR classifications for antimicrobial susceptibility assessments.

- β-lactams: ampicillin, amoxicillin-clavulanate, piperacillin-tazobactam, ceftriaxone, ceftazidime
- Carbapenems: imipenem, meropenem
- Aminoglycosides: gentamicin, tobramycin
- Fluoroquinolones: ciprofloxacin
- Others: trimethoprim/sulfamethoxazole, chloramphenicol
  1.2. Disk diffusion panels often exclude critical last-resort drugs such as: colistin, tigecycline and new β-lactam/β-lactamase inhibitor combinations.

**Response:** Dear reviewer, thank you so much for your comment. Guidelines recommend AST is typically reserved for selected high-risk clinical isolates rather than included in routine AST panels. Therefore, routine AST for colistin in the study area was limited by technical complexity (labor-intensive, time-consuming, and required trained personnel), lack of reagents, high cost and limited routine clinical demand.

1.3. Disk diffusion is not reliable for some antibiotics (e.g., colistin), where broth microdilution is the gold standard.

**Response:** Dear Reviewer, thank you for your valuable comment. Several reports have mentioned colistin as the last-resort antibiotic used for treating multidrug-resistant Gram-negative infections. Even though we did not conduct and reported colistin resistance testing through disk diffusion method in the study area due to the following reasons technical complexity (labor-intensive, time-consuming, and require trained personnel), lack of reagents and high cost.

1.4. XDR/PDR classification often benefits from minimum inhibitory concentration (MIC) values to accurately determine resistance levels.

**Response:** Dear reviewer, thank you for your valuable comment. These classifications are crucial for understanding the severity of antibiotic resistance and guiding treatment strategies. However, initial detection or screening for MDR/XDR/PDR isolates before confirmatory MIC testing conducted through a widely used disk diffusion technique in our study area.

1.5. According to international standards (e.g., ECDC), classification of XDR, and PDR requires: Testing across multiple antimicrobial classes and using validated methods for each drug class

**Response:** Dear reviewer, thank you so much for your comment. Multiple studies (Sharma R, Otieno S, Otieno S. Kirby Bauer disc diffusion method for antibiotic susceptibility testing. Microbe Notes. 2022) and CLSI M100 standards indicate that disc diffusion (Kirby-Bauer testing) for MDR/XDR/PDR determination typically involve 7–12 relevant antibiotic classes, prevents underestimating resistance and ensures reliable classification into MDR/XDR/PDR. So, we have tested 12 antibiotics for XDR and PDR classifications for antimicrobial susceptibility assessments.

2. To confidently classify isolates as XDR or PDR:
   2.1. Combine disk diffusion with: Broth microdilution, automated systems (e.g., VITEK, Phoenix) or gradient diffusion (E-test) for MIC determination.

**Response:** We sincerely thank you for your valuable comment. We agree that confidently classifying isolates as XDR or PDR ideally requires combining disk diffusion with additional methods such as broth microdilution, automated systems (e.g., VITEK, Phoenix), or gradient diffusion (E-test). Each of these methods has distinct advantages and limitations, and their selection depends on laboratory capacity and the bacterial species under investigation. However, in our study setting, limited accessibility and high costs restricted the use of these advanced methods. This constraint has been clearly acknowledged in the Limitations section of the manuscript.

2.2. Lines 131 and 132: Remove ‘Blood culture samples collection, inoculation, identification and antimicrobial susceptibility testing.

**Response:** Thank you for this insightful suggestion; we have acted on it by removing the paragraph.

2.3. Definitions (MDR, XDR and PDR) better be presented using bullet for easy understanding than a paragraph

**Response:** I truly appreciate your thoughtful feedback. Your observations provide meaningful guidance for enhancing the quality of this manuscript.

2.4. How can you analyze the AST for MDR, XDR and PDR? Which tool is used in this case?

**Response:** Analyzing Antibiotic Susceptibility Testing (AST) data to classify bacterial isolates as Multidrug-Resistant (MDR), Extensively Drug-Resistant (XDR), or Pandrug-Resistant (PDR) relies on evaluating resistance across standardized antimicrobial categories rather than individual drugs. According to the international consensus definitions established by the CDC and ECDC, an isolate is MDR if it is non-susceptible (resistant or intermediate) to at least one agent in three or more therapeutic categories. It is escalated to XDR if it remains susceptible to only one or two categories, and PDR if it is non-susceptible to every agent across all categories tested. To accurately perform this assessment, individual tested antibiotics must be mapped to their overarching classes (such as grouping Meropenem and Imipenem under Carbapenems), and a whole class is flagged as non-susceptible if even a single drug within it fails, while excluding any categories of natural, intrinsic resistance.

While raw AST data is initially generated in our laboratories through manual disk diffusion methods, WHONET software is the primary tool utilized to crunch these results into their proper resistance profiles and instantly flag MDR, XDR, and PDR isolates for public health reporting.

2.5. Avoid: This is table 2/3 legend.

**Response:** Dear reviewer, thank you so much for your comment. We have corrected table 2/3 legend based on your suggestion.

2.6. It is possible to assess the MDR, XDR or PDR for a single species, but it is totally wrong to determine/calculate the overall MDR/XDR/PDR for Gram-negatives or Gram-positives because we cannot use the same antimicrobials for all isolates. Therefore, this part should be reconsidered.

**Response:** thank you for your nice comments and suggestions. However, we can calculate Overall % prevalence as follows:

$$\text{MDR (\%)}=\frac{N_{\text{MDR}}}{N_{\text{total}}}\times100$$

$$\text{XDR (\%)}=\frac{N_{\text{XDR}}}{N_{\text{total}}}\times100$$

$$\text{PDR (\%)}=\frac{N_{\text{PDR}}}{N_{\text{total}}}\times100$$

2.7. Think of: ‘the Amhara National Regional State’ throughout the manuscript including table/figure titles.

Response: I appreciate your insightful comments about the inclusion of Amhara Public Health Institute in the article, especially the titles of the tables and figures. We included it.

2.8. Why do you consider the Addisalem General Hospital (AGH) as a reference variable the associated variables?

**Response:** I really appreciate your feedback. Addisalem General Hospital (AGH) is considered a reference variable in these studies because of its significant involvement in patient-centered care and quality management strategies.

2.9. If you mentioned some names of hospitals, why did you leave others? Were these the only hospitals that referred samples to APHI?

**Response:** We appreciate the reviewer’s observation regarding the inclusions of some hospitals only and others left. The Amhara Public Health Institute (APHI) has faced challenges in receiving blood culture samples from hospitals due to limited referrals.

2.10. The ‘discussion’ was too bulky (8 pages). It should be based on the study objective/s. It requires revision and to be targeted.

**Response:** We acknowledge the reviewer’s concern regarding the bulkiness of the discussion. In response, we have:

•	Reduced the length by eliminating non-essential details.

•	Reorganized the content to follow the sequence of the study objectives.

•	Strengthened the linkage between results and interpretation.

This targeted revision ensures that the discussion remains focused, coherent, and aligned with the purpose of the study

2.11. Excessive numbers in the discussion part should be minimized

**Response:** We acknowledge the reviewer’s concern regarding the bulkiness of the discussion.

2.12. Too many references for such a single objective and requires revision.

**Response:** We appreciate your observation regarding the number of references cited for this specific objective. We understand that excessive referencing may reduce clarity and conciseness. In response, we have carefully reviewed the cited literature and retained only the most relevant and recent sources that directly support the objective. This revision streamlines the reference list, ensuring that each citation adds clear value to the discussion without redundancy.

2.13. Lines 19, 25 & 47: Reference, not Organization, W.H. Better say WHO.

**Response:** Dear reviewer, I truly appreciate your feedback. We make amends for it.

2.14. The titles of figures should be avoided if they are already placed in the main body of the manuscript. Titles should not be embedded in figures.

**Response:** Dear reviewer, I really value your comments. we removed it.

2.15. Line 697: Reference (28); There was no article name, author, etc.

**Response:** We appreciate your comments. The article’s name, author, and other details have been added and updated in the reference (28).

**Reviewer #5:**

2.16. The revision includes meaningful improvements, particularly regarding contamination definitions and laboratory procedures. However, substantial methodological and analytical clarifications are still required.

**Response:** We appreciate the reviewer’s acknowledgment of the improvements made in defining contamination and refining laboratory procedures. We recognize the need for further methodological and analytical clarifications. In the revised manuscript, we have expanded the methodology section to provide clearer descriptions of sampling protocols, analytical techniques, and data interpretation steps.

2.17. The reported culture positivity (58.3–58.5%) remains unusually high for routine retrospective laboratory data, even in referral laboratories. Although the authors now describe contamination criteria (CDC/NHSN), important issues remain:
- Coagulase-negative staphylococci (CoNS) still represent a large proportion of isolates.
- The manuscript does not report number of culture sets per patient actually analyzed.
- proportion of isolates meeting “two consecutive cultures” requirement
- contamination rate
- proportion excluded as contaminants

**Response:** We appreciate the reviewer’s comments and suggestions. Regarding the high reported culture positivity rate (58.3–58.5%), we carefully reviewed our methodology and data collection process. The observed rate may reflect specific factors, optimal blood volume, number of culture sets, laboratory practices and patients diagnosed with sepsis tend to have higher culture positivity rates. We have clarified these points in the revised manuscript and added a discussion regarding possible reasons for the higher positivity rate and its implications in the Context and Discussion sections. We believe these revisions adequately address the concerns raised and improve the clarity of our findings.

2.18. Although the authors now describe contamination criteria (CDC/NHSN), important issues remain:
- Coagulase-negative staphylococci (CoNS) still represent a large proportion of isolates.
- number of cultures sets per patient actually analyzed
- proportion of isolates meeting “two consecutive cultures” requirement
- contamination rate
- proportion excluded as contaminants

**Response:** Various studies show that the contamination rate ranges between 0.6-6% (2-4); it depends upon the sample collection method and processing in the laboratory. The benchmark of blood contamination rate is <3%, as proposed by the Clinical Laboratory Standard Institute (CLSI) …

2.19. Could the authors report the contamination rate according to their stated criteria?

**Response**: Yes, the authors can report the contamination rate in accordance with their defined criteria.

2.20. Could the authors specify how many CoNS isolates were excluded vs retained as true pathogens? But the report must: By following these steps, the manuscript will meet standard expectations for reproducibility and transparency in microbial contamination reporting
- Clearly define sample eligibility and contaminant classification.
- Use explicit calculation methods with numerators and denominators stated.
- Provide statistical confidence intervals.
- Disclose any sampling or decontamination procedures influencing results.

**Response:** Based on the data provided in the referenced studies, the number of Coagulase-negative Staphylococci (CoNS) isolates retained as true pathogens versus those excluded as contaminants is reported as follows:

- Total 817 blood culture records retrieved
- 58.9% (N= 481) yielded growth
- Excluded (likely contaminants): 29.3 % (N=141) including Coagulase-negative staphylococci (CoNS) (27 %, N=130), *Bacillus* spp. (1.2 %, N= 6) and *Candida* spp. (1 %, N= 5).
- Remaining 676 blood culture records were included in the final analysis
- True pathogens: 50.3 % (N=340)

2.21. Identification methods remain limited

- Bacterial identification relies entirely on conventional biochemical testing.
- No confirmatory methods are reported (e.g., automated systems or advanced identification platforms).
- While resource constraints are acknowledged, this limitation must be addressed analytically.
- I suggest that the authors discuss the potential for species misidentification and its impact on resistance estimates.

**Response:** Despite the advent of many newer tests, biochemical tests are still pivotal for bacterial identification. Phenotypic and biochemical tests still hold the key for laboratory identification due to their lower cost and ease in carrying out and interpretation. Biochemical tests are the key for easily microbial identification for researchers in past, present and also in future, from various sites of infections.

2.22. Numerous grammatical and typographical errors remain.

**Response:** Thank the reviewer for your constructive comments and the time you spent reviewing this manuscript. These improvements are made through your constructive feedback.

2.23. Scientific nomenclature inconsistent (e.g., species formatting).

**Response:** Thank the reviewer for your constructive comments and we amend it.

2.24. Some abbreviations are introduced without definition.

**Response:** Thank you for pointing this out. The abbreviations have been included with their definitions where the change can be found in the revised manuscript.

2.25. Tables are difficult to interpret due to inconsistent formatting.

**Response:** We think this is an excellent suggestion. We have followed consistent formatting thought the Table and the change can be found in the revised manuscript.

2.26. Resistance percentages sometimes do not correspond clearly to denominators.

**Response:** Thank you for pointing this out. We have revised it.

2.27. Background section contains repetitive information.

**Response:** We appreciate the reviewer’s observation regarding the repetition in the background section. We have carefully revised this section to remove redundant statements and streamline the narrative. Overlapping information has been consolidated, and each paragraph now focuses on a distinct aspect of the study’s context to ensure clarity and conciseness. These changes improve the flow and avoid unnecessary reiteration while retaining essential background details for reader comprehension.

2.28. Several sentences require language editing for clarity.

**Response:** Thank you for your valuable feedback on sentence clarity. We thoroughly reviewed the given paragraphs and revised the content to improve readability, correctness, and cohesion. The amended sentences now express the required notion more directly and unambiguously.
