# Supplementary material for: Bacterial profile and antimicrobial resistance patterns of bloodstream infections at Amhara Public Health Institute, Bahir Dar, Ethiopia
Source: PLoS One. 2026 Jul 27;21(7):e0354710. doi: 10.1371/journal.pone.0354710 (PMC13405292; doi:10.1371/journal.pone.0354710)
Supplement: Peer review history

# Peer review: Decision Letter 0

**DOI:** 10.1371/journal.pone.0354710.r001

**Authors:** Mengistu Zenebe

4 Jul 2025

PONE-D-25-16842

Microbiological Profile and Antimicrobial Resistance Patterns of Blood Culture Isolates at Amhara Public Health institute: Four-year Retrospective Study

PLOS ONE

Thank you for submitting your manuscript to PLOS ONE. After careful consideration, we feel that it has merit but does not fully meet PLOS ONE’s publication criteria as it currently stands. Therefore, we invite you to submit a revised version of the manuscript that addresses the points raised during the review process.

Please submit your revised manuscript by Aug 18 2025 11:59PM. If you will need more time than this to complete your revisions, please reply to this message or contact the journal office at plosone@plos.org. When you're ready to submit your revision, log on to https://www.editorialmanager.com/pone/ and select the 'Submissions Needing Revision' folder to locate your manuscript file.

Please include the following items when submitting your revised manuscript:

- A rebuttal letter that responds to each point raised by the academic editor and reviewer(s). You should upload this letter as a separate file labeled 'Response to Reviewers'.
- A marked-up copy of your manuscript that highlights changes made to the original version. You should upload this as a separate file labeled 'Revised Manuscript with Track Changes'.
- An unmarked version of your revised paper without tracked changes. You should upload this as a separate file labeled 'Manuscript'.

If you would like to make changes to your financial disclosure, please include your updated statement in your cover letter. Guidelines for resubmitting your figure files are available below the reviewer comments at the end of this letter.

If applicable, we recommend that you deposit your laboratory protocols in protocols.io to enhance the reproducibility of your results. Protocols.io assigns your protocol its own identifier (DOI) so that it can be cited independently in the future. For instructions see: https://journals.plos.org/plosone/s/submission-guidelines#loc-laboratory-protocols. Additionally, PLOS ONE offers an option for publishing peer-reviewed Lab Protocol articles, which describe protocols hosted on protocols.io. Read more information on sharing protocols at https://plos.org/protocols?utm_medium=editorial-email&utm_source=authorletters&utm_campaign=protocols.

We look forward to receiving your revised manuscript.

Kind regards,

Mengistu Hailemariam Zenebe, PhD

Academic Editor

PLOS ONE

Journal Requirements:

When submitting your revision, we need you to address these additional requirements.

1. Please ensure that your manuscript meets PLOS ONE's style requirements, including those for file naming. The PLOS ONE style templates can be found at

https://journals.plos.org/plosone/s/file?id=wjVg/PLOSOne_formatting_sample_main_body.pdf and

https://journals.plos.org/plosone/s/file?id=ba62/PLOSOne_formatting_sample_title_authors_affiliations.pdf.

2. We note that you have indicated that there are restrictions to data sharing for this study. For studies involving human research participant data or other sensitive data, we encourage authors to share de-identified or anonymized data. However, when data cannot be publicly shared for ethical reasons, we allow authors to make their data sets available upon request. For information on unacceptable data access restrictions, please see http://journals.plos.org/plosone/s/data-availability#loc-unacceptable-data-access-restrictions.

Before we proceed with your manuscript, please address the following prompts:

a) If there are ethical or legal restrictions on sharing a de-identified data set, please explain them in detail (e.g., data contain potentially identifying or sensitive patient information, data are owned by a third-party organization, etc.) and who has imposed them (e.g., a Research Ethics Committee or Institutional Review Board, etc.). Please also provide contact information for a data access committee, ethics committee, or other institutional body to which data requests may be sent.

b) If there are no restrictions, please upload the minimal anonymized data set necessary to replicate your study findings to a stable, public repository and provide us with the relevant URLs, DOIs, or accession numbers. Please see http://www.bmj.com/content/340/bmj.c181.long for guidelines on how to de-identify and prepare clinical data for publication. For a list of recommended repositories, please see https://journals.plos.org/plosone/s/recommended-repositories. You also have the option of uploading the data as Supporting Information files, but we would recommend depositing data directly to a data repository if possible.

Please update your Data Availability statement in the submission form accordingly.

3. In the online submission form you indicate that your data is not available for proprietary reasons and have provided a contact point for accessing this data. Please note that your current contact point is a co-author on this manuscript. According to our Data Policy, the contact point must not be an author on the manuscript and must be an institutional contact, ideally not an individual. Please revise your data statement to a non-author institutional point of contact, such as a data access or ethics committee, and send this to us via return email. Please also include contact information for the third party organization, and please include the full citation of where the data can be found.

4. Please amend the manuscript submission data (via Edit Submission) to include author Yosef Gashaw.

5. We notice that your supplementary figures are uploaded with the file type 'Figure'. Please amend the file type to 'Supporting Information'. Please ensure that each Supporting Information file has a legend listed in the manuscript after the references list.

[Note: HTML markup is below. Please do not edit.]

Reviewers' comments:

Reviewer's Responses to Questions

**Comments to the Author**

1. Is the manuscript technically sound, and do the data support the conclusions?

The manuscript must describe a technically sound piece of scientific research with data that supports the conclusions. Experiments must have been conducted rigorously, with appropriate controls, replication, and sample sizes. The conclusions must be drawn appropriately based on the data presented.

Reviewer #1: Partly

Reviewer #2: Partly

**********

2. Has the statistical analysis been performed appropriately and rigorously?

Reviewer #1: I Don't Know

Reviewer #2: I Don't Know

**********

3. Have the authors made all data underlying the findings in their manuscript fully available?

The PLOS Data policy requires authors to make all data underlying the findings described in their manuscript fully available without restriction, with rare exception (please refer to the Data Availability Statement in the manuscript PDF file). The data should be provided as part of the manuscript or its supporting information, or deposited to a public repository. For example, in addition to summary statistics, the data points behind means, medians and variance measures should be available. If there are restrictions on publicly sharing data—e.g. participant privacy or use of data from a third party—those must be specified.

Reviewer #1: No

Reviewer #2: Yes

**********

4. Is the manuscript presented in an intelligible fashion and written in standard English?

PLOS ONE does not copyedit accepted manuscripts, so the language in submitted articles must be clear, correct, and unambiguous. Any typographical or grammatical errors should be corrected at revision, so please note any specific errors here.

Reviewer #1: No

Reviewer #2: No

**********

5. Review Comments to the Author

Please use the space provided to explain your answers to the questions above. You may also include additional comments for the author, including concerns about dual publication, research ethics, or publication ethics. (Please upload your review as an attachment if it exceeds 20,000 characters)

Reviewer #1: The study is valuable in identifying the common pathogens of bloodstream infection as well as their antimicrobial resistance pattern. However, corrections are highly recommended.

Corrections required:

In general:

1. Scientific names of organisms are not in italics.

2. The “spp.” need not be in italics.

3. Digits after decimal point should be uniform.

In abstract:

1. It is mentioned that “..all aged groups..” were included. However, the main article shows not only aged people, but a wide range of patients were included in the study.

2. Why was Gram staining of blood done? It is redundant to diagnose infection.

3. Only 24 hours of incubation of the specimen is mentioned which is inadequate for blood culture.

In main article:

1. Line 69: Correction of “E.colli” is required.

2. Line 71: It should be BSI, not BIS.

3. Line 129: As per Oxoid website, their product is tryptone soya broth, not tryptic soya broth. Confirmation of the product used is recommended.

4. Line 133: Why was the range of 35 to 37oC kept for incubation? Was there any reason for changing the temperature during actual work? Please specify.

5. Line 139: Only MacConkey is mentioned. Whether agar or broth is missing.

6. Lines 149-151: Identification of Enterococcus is missing.

7. Line 154: Why was normal saline used to incubation instead of broth? Any references for this method of AST?

8. Line 159: Spelling is Mueller-Hinton agar, not Muller-Hinton agar.

9. Line 201: ATCC should not be within brackets.

10. Line 205-208: The sentence is unclear.

11. Lines 310-312: Resistance to number of antibiotics is specified in table as R1, R2, etc. It does not fit the MDR criteria.

Reviewer #2: Dear Editor,

Thank you for your invitation to review the manuscript titled “Microbiological Profile and Antimicrobial Resistance Patterns of Blood Culture Isolates at Amhara Public Health institute: Four-year Retrospective Study.” I have provided my comments for each section of the paper.

• Title: It is better modified as, Bacterial Profile and Antimicrobial Resistance Patterns of Blood Culture Isolates at Amhara Public Health institute: Four-year Retrospective Study. It was more about bacteria. The author/s mentioned at once ‘Candida species. And this cannot qualify the status of ‘microbiological profile’. Even microbiological Profile by itself is too broad.

Abstract

• Line 25: Requires revision. For example, ‘Therefore, this study was to analyze the microbiological profile and antimicrobial resistant patterns among patients……….’ It is better written as, ‘Therefore, this study was intended to assess the bacterial profile and antimicrobial resistant patterns among patients……………..

• In the method part of the abstract, first describe the laboratory techniques than encoding to excel or SPSS. Even when you write the lab technique, it seems as you did prospectively. You have to make it compatible to retrospective study. The study was done in a reference laboratory, but you mentioned as you did preliminary test. Please think over it.

• Line 37: not cases. It is better written as, ‘Of them, 267 (55.5%) isolates were Gram-positive, 209 (43.4%) isolates Gram-negative bacteria, and the remaining 5 (1.1%) were Candida species.’

• Line 39: What does ‘ESKAPE’ represent? It should be written in both the long and abbreviated form as its first appearance in combination.

• Line 43: No italics is needed for ‘Coagulase-negative staphylococci’

• Line 44: You can calculate the overall prevalence of each bacterium, but it is impossible to calculate the overall prevalence. For example, based on CLSI guideline, we cannot test the same antibiotics for all isolates. You have to revise it and all the result parts.

Introduction

• The rationale is not explained well. Why you did this research? What has been done before, what is known before and which you intend to fill? The introduction should be coherent and interlinked.

Materials and methods

• The method part should be written in standard English.

• What were the inclusions and exclusion criteria? Have you collected any of what you got?

• Lines 159&166: not Muller Hinton agar, rather ‘Mueller Hinton agar’

Result

• The result should be revised and written well.

• In ‘the associated factors’ part the reference selection was not logical. For example, why you select, ‘Addis Alem General Hospital’ as a reference?

• Specific comments will be forwarded if the paper turns back to me.

Discussion

• Discussion not merely comparison, but it includes justifications of your results

• Try to decrease numbers in the discussion part.

• Your conclusion and recommendation were too ambitious. It should be specific and based on your results.

• Figures and tables should be prepared to the standard.

• The references were not written properly. All references should be written correctly according to Vancouver style.

• Follow the standard binomial nomenclature, italize journal name and the word ‘et al’

• Follow the guideline for manuscript writing protocol for PLoS One.

**********

6. PLOS authors have the option to publish the peer review history of their article (what does this mean?). If published, this will include your full peer review and any attached files.

If you choose “no”, your identity will remain anonymous but your review may still be made public.

**Do you want your identity to be public for this peer review?**  For information about this choice, including consent withdrawal, please see our Privacy Policy.

Reviewer #1: No

Reviewer #2: No

**********

[NOTE: If reviewer comments were submitted as an attachment file, they will be attached to this email and accessible via the submission site. Please log into your account, locate the manuscript record, and check for the action link "View Attachments". If this link does not appear, there are no attachment files.]

While revising your submission, please upload your figure files to the Preflight Analysis and Conversion Engine (PACE) digital diagnostic tool, https://pacev2.apexcovantage.com/. PACE helps ensure that figures meet PLOS requirements. To use PACE, you must first register as a user. Registration is free. Then, login and navigate to the UPLOAD tab, where you will find detailed instructions on how to use the tool. If you encounter any issues or have any questions when using PACE, please email PLOS at figures@plos.org. Please note that Supporting Information files do not need this step.

Attachment Submitted filename: Review file.pdf

# Author comment: Author response to Decision Letter 1

**DOI:** 10.1371/journal.pone.0354710.r002

25 Aug 2025

PONE-D-25-16842

Title: Bacterial profile and antimicrobial resistance patterns of blood culture isolates at Amhara Public Health Institute, Bahirdar, Ethiopia: Four-year retrospective study

Dear Academic Editor PLOS ONE and Reviewers,

We would like to begin by thanking the editor and reviewers for their time spent revewing this manuscript and for their many helpful comments. We have addressed all the suggestions in our current version and we believe the manuscript has been improved. In this letter, our responses are written following each suggesions and questions. We have included a marked-up copy (word) of the manuscript that highlights changes made to the original version are upload as a separate file labeled 'Revised Manuscript with Track Changes and unmarked version of our revised paper without tracked changes are upload as a separate file labeled 'Manuscript'.

Sincerely,

Michael Getie ( Corresponding author)

Attachment Submitted filename: Response to Reviewers.docx

# Peer review: Decision Letter 1

**DOI:** 10.1371/journal.pone.0354710.r003

**Authors:** Mengistu Zenebe

6 Oct 2025

PONE-D-25-16842R1

Bacterial profile and antimicrobial resistance patterns of blood culture isolates at Amhara Public Health Institute, Bahirdar, Ethiopia: Four-year retrospective study

PLOS ONE

Thank you for submitting your manuscript to PLOS ONE. After careful consideration, we feel that it has merit but does not fully meet PLOS ONE’s publication criteria as it currently stands. Therefore, we invite you to submit a revised version of the manuscript that addresses the points raised during the review process.

Please submit your revised manuscript by Nov 20 2025 11:59PM. If you will need more time than this to complete your revisions, please reply to this message or contact the journal office at plosone@plos.org. When you're ready to submit your revision, log on to https://www.editorialmanager.com/pone/ and select the 'Submissions Needing Revision' folder to locate your manuscript file.

Please include the following items when submitting your revised manuscript:

- A rebuttal letter that responds to each point raised by the academic editor and reviewer(s). You should upload this letter as a separate file labeled 'Response to Reviewers'.
- A marked-up copy of your manuscript that highlights changes made to the original version. You should upload this as a separate file labeled 'Revised Manuscript with Track Changes'.
- An unmarked version of your revised paper without tracked changes. You should upload this as a separate file labeled 'Manuscript'.

If you would like to make changes to your financial disclosure, please include your updated statement in your cover letter. Guidelines for resubmitting your figure files are available below the reviewer comments at the end of this letter.

If applicable, we recommend that you deposit your laboratory protocols in protocols.io to enhance the reproducibility of your results. Protocols.io assigns your protocol its own identifier (DOI) so that it can be cited independently in the future. For instructions see: https://journals.plos.org/plosone/s/submission-guidelines#loc-laboratory-protocols. Additionally, PLOS ONE offers an option for publishing peer-reviewed Lab Protocol articles, which describe protocols hosted on protocols.io. Read more information on sharing protocols at https://plos.org/protocols?utm_medium=editorial-email&utm_source=authorletters&utm_campaign=protocols.

We look forward to receiving your revised manuscript.

Kind regards,

Mengistu Hailemariam Zenebe, PhD

Academic Editor

PLOS ONE

Journal Requirements:

If the reviewer comments include a recommendation to cite specific previously published works, please review and evaluate these publications to determine whether they are relevant and should be cited. There is no requirement to cite these works unless the editor has indicated otherwise.

[Note: HTML markup is below. Please do not edit.]

Reviewers' comments:

Reviewer's Responses to Questions

**Comments to the Author**

1. If the authors have adequately addressed your comments raised in a previous round of review and you feel that this manuscript is now acceptable for publication, you may indicate that here to bypass the “Comments to the Author” section, enter your conflict of interest statement in the “Confidential to Editor” section, and submit your "Accept" recommendation.

Reviewer #2: All comments have been addressed

Reviewer #3: (No Response)

Reviewer #4: (No Response)

**********

2. Is the manuscript technically sound, and do the data support the conclusions?

The manuscript must describe a technically sound piece of scientific research with data that supports the conclusions. Experiments must have been conducted rigorously, with appropriate controls, replication, and sample sizes. The conclusions must be drawn appropriately based on the data presented.

Reviewer #2: Yes

Reviewer #3: Yes

Reviewer #4: Partly

**********

3. Has the statistical analysis been performed appropriately and rigorously?

Reviewer #2: Yes

Reviewer #3: Yes

Reviewer #4: No

**********

4. Have the authors made all data underlying the findings in their manuscript fully available?

The PLOS Data policy requires authors to make all data underlying the findings described in their manuscript fully available without restriction, with rare exception (please refer to the Data Availability Statement in the manuscript PDF file). The data should be provided as part of the manuscript or its supporting information, or deposited to a public repository. For example, in addition to summary statistics, the data points behind means, medians and variance measures should be available. If there are restrictions on publicly sharing data—e.g. participant privacy or use of data from a third party—those must be specified.

Reviewer #2: Yes

Reviewer #3: Yes

Reviewer #4: No

**********

5. Is the manuscript presented in an intelligible fashion and written in standard English?

PLOS ONE does not copyedit accepted manuscripts, so the language in submitted articles must be clear, correct, and unambiguous. Any typographical or grammatical errors should be corrected at revision, so please note any specific errors here.

Reviewer #2: Yes

Reviewer #3: Yes

Reviewer #4: No

**********

6. Review Comments to the Author

Please use the space provided to explain your answers to the questions above. You may also include additional comments for the author, including concerns about dual publication, research ethics, or publication ethics. (Please upload your review as an attachment if it exceeds 20,000 characters)

Reviewer #2: All the comments were adressed well. The declaration section needs improvement during at/or before proof reading, specially authors contribtion should be similar to the submision systems'.

Reviewer #3: Dear Editor

This manuscript is about " Bacterial profile and antimicrobial resistance patterns of blood culture isolates at

Amhara Public Health Institute, Bahirdar, Ethiopia: Four-year retrospective study". It is a good manuscript but it is not suitable for publishing in PLOS ONE. This study is just a simple report of bacterial blood culture and the frequency of their antibiotic resistant. So, It can not catch the eye of your readers. So , I decided to reject it.

Regards,

Reviewer #4: This is regarding a manuscript titled Bacterial profile and antimicrobial resistance patterns of blood culture isolates at Amhara Public Health Institute, Bahirdar, Ethiopia: Four-year retrospective study

General comment

This manuscript narrates the Bacterial profile and antimicrobial resistance patterns of blood culture isolates. The study design is thorough and contextualizes their findings within the urgent national issue of antibiotic resistance in connection with BSI.

At the same time, the work repeats a familiar approach without advancing new methodologies for the identification of bacteria and drug resistance. Speciation of many bacteria and Candida is not done. Besides, BSI-causing anaerobic bacteria are not included. The English part of the manuscript needs to be revised.

Recommendations

The title should be modified

Bacterial profile and antimicrobial resistance patterns of bloodstream infections at Amhara Public Health Institute, Bahirdar, Ethiopia: Four-year retrospective study

Abstract

1. The abstract should be improved

2. Replace specimen with samples throughout the manuscript

3. Line 34 The way the overall culture positive findings are presented in the results is not logical. It should include both bacteria and Yeast

4. Standard bacteriological techniques were followed during blood collection. What does it mean? Have authors followed standardized manual blood culture methods following WHO recommendations for resource-limited settings?

5. Techniques used for the AMST test have to be mentioned

6. Is this a work intended to show the problems of ESKAPE pathogens in BSI

7. How about Community-onset BSIs

8. What is the criteria for including patients for Nosocomial BSI with ESKAPE pathogens

9. The acronym has now been revised as ESKAPEE. So update the results

10. Why didn’t the authors include the WHO priority pathogens in their study?

11. Results of the overall antibiogram profile have to be included, rather than focusing only on ESKAPE pathogens. Results of drug resistance have to be revised by including the names of antibiotics versus the percentage of resistance

12. Avoid repetitive citation of the abbreviation on lines 24 and 47

13. Use the microbiological rule of abbreviating bacteria. Lines 39 and 40

14. Parentheses have to be standardized. N should be placed inside the bracket

15. If P values are reported, please follow standard conventions for decimal places: for P values less than .001, report "P<.001"; for P values between .001 and .01, report the value to the nearest thousandth; for P values greater than or equal to .01, report the value to the nearest hundredth; and for P values greater than .99, report as "P>.99."

16. In conclusion, the authors mentioned the carbapenem resistance without indicating any findings in the results session

17. What about recommending an antimicrobial stewardship programe?

18. Is there any existing antimicrobial stewardship programe in the study setting?

Introduction

It is too general

Clearly state the problem, causes, and outcomes of BSI

Include details of BSI-associated morbimortality in Ethiopia

What type of Research gap exists in the study area?

Include some lines on how BSI infection is treated/managed in Ethiopia

Methods

Study area: Remove irrelevant information for international readers from lines 102 to 109.

Should include cases and fatality related to BSI in terms of morbimortality has to be introduced in the study setting. Standard treatment guidelines of BSI in Ethiopia

Bloodstream infections should be detected through standardized manual blood culture methods following WHO recommendations for resource-limited settings. There is no reference in this regard. Factors that may influence the recovery of pathogens from the blood include the timing of blood collection, the number of sets collected, and blood volume.

True BSIs should be distinguished from potential contaminants (e.g., Micrococcus spp., Bacillus spp. Other than B. anthracis, or coagulase-negative staphylococci (CoNS) using standardized definitions adapted from the CDC guidelines. However, this is missing in this study.

Why this study excludes Community-onset BSIs ?

How only ESKAPE pathogens associated BSI is highlighted?

What is the overall prevalence of ESKAPE pathogens in the study settings

Is there any data related to the upsurge of ESKAPE pathogens in the hospital?

Is there any local antibiotic policy used in the hospital to manage ESKAPE pathogens?

In the results, authors mentioned Candida. However, methodology for its isolation and identification are missing.

What is the selection criteria for antibiotics?

Accurate identification of bacterial species and susceptibility testing is important, but I am not convinced that they have accurately identified the species level of the isolates in their study.

Staphylococcus (CONS), Proteus and Klebsiella are heterogeneous group of pathogenic organisms. It is not acceptable to lump them together, considering the objectives of the study.

The authors are advised to classify the tested isolates to MDR , XDR, and PDR as described by Magiorakos et al.

Why authors didn’t inspected MRSA, since these are the WHO prioritized drug resistant bacteria, since the prevalence of these bacteria are frequently appearing in the papers from Ethiopia.

Why authors didn’t checked the presence of extensive drug resistance (XDR) and pan-drug resistance (PDR), even though the MDR is 68%.

Details of the vital signs of patients have to be included?

Predetermined sample size calculation is missing

Precisely include the eligibility criteria

The details statistical analysis done are vague. Include all variables regardless of their p-value in the univariate analysis to avoid missing important confounders.

Strengthen the discussion. Deepen the critical analysis by comparing findings with other recent plant-based antimicrobial studies (especially >2020).

Discuss limitations more transparently

**********

7. PLOS authors have the option to publish the peer review history of their article (what does this mean?). If published, this will include your full peer review and any attached files.

If you choose “no”, your identity will remain anonymous but your review may still be made public.

**Do you want your identity to be public for this peer review?**  For information about this choice, including consent withdrawal, please see our Privacy Policy.

Reviewer #2: No

Reviewer #3: No

Reviewer #4: **Yes:** ASEER MANILAL

**********

[NOTE: If reviewer comments were submitted as an attachment file, they will be attached to this email and accessible via the submission site. Please log into your account, locate the manuscript record, and check for the action link "View Attachments". If this link does not appear, there are no attachment files.]

While revising your submission, please upload your figure files to the Preflight Analysis and Conversion Engine (PACE) digital diagnostic tool, https://pacev2.apexcovantage.com/. PACE helps ensure that figures meet PLOS requirements. To use PACE, you must first register as a user. Registration is free. Then, login and navigate to the UPLOAD tab, where you will find detailed instructions on how to use the tool. If you encounter any issues or have any questions when using PACE, please email PLOS at figures@plos.org. Please note that Supporting Information files do not need this step.

# Author comment: Author response to Decision Letter 2

**DOI:** 10.1371/journal.pone.0354710.r004

21 Nov 2025

Dear Dr. Mengistu,

PhD Academic Editor

PLOS ONE

Authors' response to reviewers' comments:

Reviewer #2: All the comments were addressed well. The declaration section needs improvement during/or before proofreading, especially the author's contribution should be similar to the submission systems.

Replay: Dear reviewer, many thanks for your remark. The declaration section, which primarily addresses the author's contribution, has been updated in response to your comments.

Reviewer #3: Dear Editor

This manuscript is about " Bacterial profile and antimicrobial resistance patterns of blood culture isolates at Amhara Public Health Institute, Bahirdar, Ethiopia: Four-year retrospective study". It is a good manuscript, but it is not suitable for publishing in PLOS ONE. This study is just a simple report of bacterial blood culture and the frequency of their antibiotic resistant.

Replay: Dear reviewer, I really value your comments. Bloodstream infection-related antimicrobial resistance is a global concern, yet, managing blood stream infection in Ethiopia is hampered by lack of bacteriological support needed for antimicrobial stewardship, and background data needed for empirical treatment. In addition, diagnostic culture laboratories for evaluating antimicrobial resistance are intermittent due to resources constraints, poor microbiology laboratory infrastructure, and a shortage of trained personnel. To help with life-saving clinical decisions, the clinical Laboratory Standards Institute (CLSI) recommended antimicrobials selected depending on bacterial profile, antimicrobial resistance patterns and the site of infection. To help clinicians find alternative treatments for managing bloodstream infections, this study will give them up-to-date information. It will also help other relevant entities evaluate and implement antibiotic resistance caused by bloodstream infection prevention and control strategies.

Reviewer #4: This manuscript narrates the Bacterial profile and antimicrobial resistance patterns of blood culture isolates. The study design is thorough and contextualizes their findings within the urgent national issue of antibiotic resistance in connection with BSI. At the same time, the work repeats a familiar approach without advancing new methodologies for the identification of bacteria and drug resistance. Speciation of many bacteria and Candida is not done. Besides, BSI-causing anaerobic bacteria are not included. The English part of the manuscript needs to be revised.

Replay: Dear reviewer, thank you so much for your comment. This is a retrospective study that provides updated information to the concerned body and encourages the scientific community to carry out further research in this area. Blood culture isolates, including both bacterial and fungal isolates, were identified by conventional methods to genus and species level. However, to provide comprehensive information on resistance patterns, lump them together.

Recommendations

The title should be modified

Bacterial profile and antimicrobial resistance patterns of bloodstream infections at Amhara Public Health Institute, Bahirdar, Ethiopia: Four-year retrospective study

Replay: Dear reviewer, thank you so much for your comment. We have corrected the title based on your suggestion.

Abstract

1. The abstract should be improved

Replay: Dear reviewer, thank you so much for your comment. We have properly revised the abstract section.

2. Replace specimen with samples throughout the manuscript

Replay: Dear reviewer, we corrected based on your suggestions. However, a sample is one or more parts taken from a specimen. However, a specimen is a discrete portion of blood taken from the human body for examination, analysis, or study (ISO 15189 Fourth edition 2022)

3. Line 34 The way the overall culture's positive findings are presented in the results is not logical.

Replay: Dear reviewer, thank you very much for your feedback. It has been fixed.

4. Standard bacteriological techniques were followed during blood collection. What does it mean? Have authors followed standardized manual blood culture methods following WHO recommendations for resource-limited settings?

Replay: Dear reviewer, I appreciate your kind suggestions very much. I am referring to using WHO-recommended standardized manual blood culture techniques in areas with limited resources.

5. Techniques used for the AST test have to be mentioned

Replay: We appreciate your kind words. We included it.

6. Is this a work intended to show the problems of ESKAPE pathogens in BSI

Replay: Dear reviewer, thanks for your input. This study highlights that the most predominant bacteria isolated in BSIs are the ESKAPE bacteria, which are a major concern due to their multidrug resistance (MDR) profiles and their relevance in nosocomial settings. The Infectious Diseases Society of America coined the acronym ESKAPE to emphasize these pathogens’ ability to escape or evade common therapies through antimicrobial resistance (1).

7. How about Community-onset BSIs

Replay: Thank you for your kind inquiries. Instead of community-onset BSIs, this study predicts hospital-acquired infections, BSIs, and other illnesses based on a patient's clinical history.

8. What is the criteria for including patients for Nosocomial BSI with ESKAPE pathogens

Replay: Dear reviewer, thank you for your kind remarks. Based on symptoms and clinical presentation, Nosocomial BSI is considered; the infection cannot be present at admission; rather, it must develop at least 48 hours after admission.

9. The acronym has now been revised as ESKAPEE. So update the results

Replay: We are grateful for your warm remarks. We have included and updated the results. ESKAPE is an acronym comprising the scientific names of six highly virulent and antibiotic-resistant bacterial pathogens, including: Enterococcus spp, Staphylococcus aureus, Klebsiella pneumoniae, Acinetobacter baumannii, Pseudomonas aeruginosa, and Enterobacter spp. The acronym is sometimes extended to ESKAPEE to include E.coli.

10. Why didn’t the authors include the WHO priority pathogens in their study?

Replay: Dear reviewer, twenty-four pathogens, representing fifteen families of antibiotic-resistant bacterial pathogens, are listed as WHO priority pathogens for 2024. Acinetobacter bumannaii, Pseudomonas aeruginosa, Klebsiella pneumonia, E. Coli, Enterococci, Staphylococcus aureus, and other notable bacteria were categorized as important and high-priority bacterial isolates (2). So, our study included WHO priority pathogens.

11. Results of the overall antibiogram profile have to be included, rather than focusing only on ESKAPE pathogens. Results of drug resistance have to be revised by including the names of antibiotics versus the percentage of resistance

Replay: Dear reviewer, we appreciate your insightful remarks. We included an overall antibiogram profile of both Gram-positive and Gram-negative bacterial isolates.

12. Avoid repetitive citation of the abbreviation on lines 24 and 47

Replay: Dear reviewer, We have made changes.

13. Use the microbiological rule of abbreviating bacteria. Lines 39 and 40

Replay: Dear reviewer, we appreciate your kind words. We have fixed it.

14. Parentheses have to be standardized. N should be placed inside the bracket

Replay: Dear reviewer, we appreciate your kind words. We have corrected it.

15. If P values are reported, please follow standard conventions for decimal places: for P values less than .001, report "P<.001"; for P values between .001 and .01, report the value to the nearest thousandth; for P values greater than or equal to .01, report the value to the nearest hundredth; and for P values greater than .99, report as "P>.99."

Replay: Dear reviewer, we appreciate your kind words. We have corrected it.

16. In conclusion, the authors mentioned the carbapenem resistance without indicating any findings in the results session

Replay: Dear reviewer, we value your opinions. We have corrected it.

17. What about recommending an antimicrobial stewardship programe?

Replay: Dear reviewer, Hospitals and other healthcare facilities in the Amhara region have an antimicrobial stewardship program, however, it is not fully functional and not consist of core members like microbiologists. This limits the ability to detect antibiotic resistance in BSI infections and select appropriate treatment management alternatives.

18. Is there any existing antimicrobial stewardship programe in the study setting?

Replay: Dear reviewer, the Amhara Public Health Institute does not have an AMS program, but it does serve as a coordinating regional center for AMS and AMR-related programs.

Introduction

It is too general

Clearly state the problem, causes, and outcomes of BSI

Include details of BSI-associated morbidity in Ethiopia

What type of Research gap exists in the study area?

Include some lines on how BSI infection is treated/managed in Ethiopia

Replay: Dear reviewer, thank you so much for your comment. We have revised introduction part based on given comments.

Methods

Study area: Remove irrelevant information for international readers from lines 102 to 109.

Should include cases and fatality related to BSI in terms of morbimortality has to be introduced in the study setting. Standard treatment guidelines of BSI in Ethiopia

Bloodstream infections should be detected through standardized manual blood culture methods following WHO recommendations for resource-limited settings. There is no reference in this regard. Factors that may influence the recovery of pathogens from the blood include the timing of blood collection, the number of sets collected, and blood volume.

Replay: Dear reviewer, thank you so much for your comment. We have revised methods

part based on given comments.

True BSIs should be distinguished from potential contaminants (e.g., Micrococcus spp., Bacillus spp. Other than B. anthracis, or coagulase-negative staphylococci (CoNS) using standardized definitions adapted from the CDC guidelines. However, this is missing in this study.

Replay: Dear reviewer, thank you so much for your comment. We have a standard operating procedure for manual blood culture methods to rule out which were true priority pathogen or potential contaminants. For common skin contaminants, the number of positive cultures may be used as a means of differentiating contamination from bacteremia. However, most of the published literature in this regard has focused on hospital-onset coagulase-negative staphylococcal infections. For this reason, we have not perform AST for all isolated organisms including CONS (N=128) and Bacillus spp (N=6) considered as contaminates. Additionally, included operational definitions for both true priority pathogen and potential contaminants.

Why this study excludes Community-onset BSIs ?

Replay: Dear reviewer, thank you so much for your comment. We did not excluded patients with community-onset BSIs, fungal BSI and all patients with hospitalization <48 hours. In this study patients clinical history predicts to BSIs, hospital-acquired and other infections rather than Community-onset BSIs .The inclusion criteria were suspicion of BSIs; complete records including demographic variables, clinical setting, clinical history, previous antimicrobial use, year of diagnosis, blood culture identification and AST results and presentation at any of the following inpatient or outpatient units.

How only ESKAPE pathogens associated BSI is highlighted?

Replay: Dear reviewer, thank you so much for your comment. ESKAPE pathogens are a major cause of bloodstream infections (BSI), accounting for a significant proportion of cases, with our studies reporting prevalence rates of 64.3 % (N=306) of all BSI isolates in patients with suspicion of BSIs. Their ability to "escape" the effects of common antibiotics highlight a serious global health threat, leading to higher healthcare costs, longer hospital stays, and increased mortality compared to non-ESKAPE infections (3) .

What is the overall prevalence of ESKAPE pathogens in the study settings

Replay: Dear reviewer, in this study overall prevalence ESKAPE pathogens were 64.3 % (N=306).

Is there any data related to the upsurge of ESKAPE pathogens in the hospital?

Replay: Dear reviewers, thank you so much for your comment. There are evidence support the upsurage of ESKAPE pathogens in hospitals in Ethiopia, Felege Hiwot Comprehensive Specialized Hospital 67%(N=1667), Arba Minch General Hospital 65.3% (N=207) and hospitals in Romania 97% (N=4164) of the isolates.

Is there any local antibiotic policy used in the hospital to manage ESKAPE pathogens?

Replay: Dear reviewer, thank you so much for your comment. There were compressive local antibiotics polices in health facilities /hospitals to curb the spread of ESKAPE pathogens. However antibiotic policy in a health-care setting couldn’t not efficient due to a number of factors, lack commitment of top management, absence of multidisciplinary antibiotic management team with 6–10 members with expertise and experience in different subjects (usually infectious diseases, internal medicine, surgery, pediatrics, clinical microbiology, pharmacology and hospital pharmacy), skills gap to conduct literature and systematic reviews and lack of inputs from all stakeholders in order to ensure “ownership” of guidelines.

In the results, authors mentioned Candida. However, methodology for its isolation and identification are missing.

Replay: Dear reviewer, thank you so much for your comment. Candidemia is a leading cause of healthcare-associated BSI, with all-cause in-hospital mortality reaching 30% in the United States (Toda M et al, 2019). We have included isolation and identification techniques.

What is the selection criterion for antibiotics?

Replay: Dear reviewer, thank you so much for your comment. Antibiotics were selected based on specimen type and types of infection using CLSI 2024 M 100 ED 34.

Accurate identification of bacterial species and susceptibility testing is important, but I am not convinced that they have accurately identified the species level of the isolates in their study.

Replay: Dear reviewer, thank you so much for raising your concerns. However blood specimen collection, identification, and antimicrobial susceptibility testing for this study were conducted at Amhara Public Health Institute of Bacteriology Reference Laboratory, which was accredited with full scope for a consecutive four years by (ENAO).

Staphylococcus (CONS), Proteus and Klebsiella are heterogeneous group of pathogenic organisms. It is not acceptable to lump them together, considering the objectives of the study.

Replay: Dear reviewer, I appreciate your comment. I didn't group them together. But Proteus species did not isolate in our study.

The authors are advised to classify the tested isolates to MDR , XDR, and PDR as described by Magiorakos et al.

Replay: Dear reviewer, considering your input, we have included it.

Why authors didn’t inspected MRSA, since these are the WHO prioritized drug resistant bacteria, since the prevalence of these bacteria are frequently appearing in the papers from Ethiopia.

Replay: Dear reviewer, I greatly appreciate your comment. We reported that the Cefoxitin disc (30 μg), a serogate marker for oxacillin, was evaluated against Staphylococcus aureus. It is reported that if oxacillin is resistant, it is also resistant to methicillin.

Why authors didn’t check the presence of extensive drug resistance (XDR) and pan-drug resistance (PDR), even though the MDR is 68%.

Replay: Dear reviewer, considering your input, we have included it.

Details of the vital signs of patients have to be included?

Replay: Dear reviewer, to inform you that this studies information was generated from blood culture test orders for initial treatment management of patients. Secondly analyses conducted for research purpose. We could not find details vital signs of patients in the bacteriology reference laboratory during data collection.

Predetermined sample size calculation is missing

Replay: Dear reviewer, in retrospective studies, sample size calculation can be somewhat challenging but is not impossible; it is due to the natu

Attachment Submitted filename: Response_to_Reviewers_auresp_2.docx

# Peer review: Decision Letter 2

**DOI:** 10.1371/journal.pone.0354710.r005

**Authors:** Mengistu Zenebe

27 Apr 2026

PONE-D-25-16842R2

Bacterial profile and antimicrobial resistance patterns of bloodstream infections at Amhara Public Health Institute, Bahirdar, Ethiopia

PLOS One

Dear Author,

Thank you for submitting your manuscript to PLOS ONE. After careful consideration, we feel that it has merit but does not fully meet PLOS ONE’s publication criteria as it currently stands. Therefore, we invite you to submit a revised version of the manuscript that addresses the points raised during the review process.

Please submit your revised manuscript by Jun 11 2026 11:59PM. If you will need more time than this to complete your revisions, please reply to this message or contact the journal office at plosone@plos.org. When you're ready to submit your revision, log on to https://www.editorialmanager.com/pone/ and select the 'Submissions Needing Revision' folder to locate your manuscript file.

Please include the following items when submitting your revised manuscript:

- A letter that responds to each point raised by the academic editor and reviewer(s). You should upload this letter as a separate file labeled 'Response to Reviewers'.
- A marked-up copy of your manuscript that highlights changes made to the original version. You should upload this as a separate file labeled 'Revised Manuscript with Track Changes'.
- An unmarked version of your revised paper without tracked changes. You should upload this as a separate file labeled 'Manuscript'.

If you would like to make changes to your financial disclosure, please include your updated statement in your cover letter. Guidelines for resubmitting your figure files are available below the reviewer comments at the end of this letter.

If applicable, we recommend that you deposit your laboratory protocols in protocols.io to enhance the reproducibility of your results. Protocols.io assigns your protocol its own identifier (DOI) so that it can be cited independently in the future. For instructions see: https://journals.plos.org/plosone/s/submission-guidelines#loc-laboratory-protocols. Additionally, PLOS ONE offers an option for publishing peer-reviewed Lab Protocol articles, which describe protocols hosted on protocols.io. Read more information on sharing protocols at https://plos.org/protocols?utm_medium=editorial-email&utm_source=authorletters&utm_campaign=protocols.

As the corresponding author, your ORCID iD is verified in the submission system and will appear in the published article. PLOS supports the use of ORCID, and we encourage all coauthors to register for an ORCID iD and use it as well. Please encourage your coauthors to verify their ORCID iD within the submission system before final acceptance, as unverified ORCID iDs will not appear in the published article. *Only*  the individual author can complete the verification step; PLOS staff *cannot*  verify ORCID iDs on behalf of authors.

We look forward to receiving your revised manuscript.

Kind regards,

Mengistu Hailemariam Zenebe, PhD

Academic Editor

PLOS One

Journal Requirements:

If the reviewer comments include a recommendation to cite specific previously published works, please review and evaluate these publications to determine whether they are relevant and should be cited. There is no requirement to cite these works unless the editor has indicated otherwise.

Please review your reference list to ensure that it is complete and correct. If you have cited papers that have been retracted, please include the rationale for doing so in the manuscript text, or remove these references and replace them with relevant current references. Any changes to the reference list should be mentioned in the rebuttal letter that accompanies your revised manuscript. If you need to cite a retracted article, indicate the article’s retracted status in the References list and also include a citation and full reference for the retraction notice.

[Note: HTML markup is below. Please do not edit.]

Reviewers' comments:

Reviewer's Responses to Questions

**Comments to the Author**

1. If the authors have adequately addressed your comments raised in a previous round of review and you feel that this manuscript is now acceptable for publication, you may indicate that here to bypass the “Comments to the Author” section, enter your conflict of interest statement in the “Confidential to Editor” section, and submit your "Accept" recommendation.

Reviewer #2: (No Response)

Reviewer #5: (No Response)

**********

2. Is the manuscript technically sound, and do the data support the conclusions?

The manuscript must describe a technically sound piece of scientific research with data that supports the conclusions. Experiments must have been conducted rigorously, with appropriate controls, replication, and sample sizes. The conclusions must be drawn appropriately based on the data presented.

Reviewer #2: Partly

Reviewer #5: Yes

**********

3. Has the statistical analysis been performed appropriately and rigorously?

Reviewer #2: No

Reviewer #5: Yes

**********

4. Have the authors made all data underlying the findings in their manuscript fully available?

The PLOS Data policy requires authors to make all data underlying the findings described in their manuscript fully available without restriction, with rare exception (please refer to the Data Availability Statement in the manuscript PDF file). The data should be provided as part of the manuscript or its supporting information, or deposited to a public repository. For example, in addition to summary statistics, the data points behind means, medians and variance measures should be available. If there are restrictions on publicly sharing data—e.g. participant privacy or use of data from a third party—those must be specified.

Reviewer #2: Yes

Reviewer #5: Yes

**********

5. Is the manuscript presented in an intelligible fashion and written in standard English?

PLOS ONE does not copyedit accepted manuscripts, so the language in submitted articles must be clear, correct, and unambiguous. Any typographical or grammatical errors should be corrected at revision, so please note any specific errors here.

Reviewer #2: No

Reviewer #5: No

**********

6. Review Comments to the Author

Please use the space provided to explain your answers to the questions above. You may also include additional comments for the author, including concerns about dual publication, research ethics, or publication ethics. (Please upload your review as an attachment if it exceeds 20,000 characters)

Reviewer #2: • Bahir Dar, not Bahirdar

1. The disk diffusion method (Kirby–Bauer) is useful for routine AST, but it has limitations when classifying advanced resistance categories like XDR and PDR:

• XDR and PDR definitions require testing against all or nearly all relevant antimicrobial categories.

• Disk diffusion panels often exclude critical last-resort drugs such as: colistin, tigecycline and new β-lactam/β-lactamase inhibitor combinations

• Disk diffusion is not reliable for some antibiotics (e.g., colistin), where broth microdilution is the gold standard.

• XDR/PDR classification often benefits from minimum inhibitory concentration (MIC) values to accurately determine resistance levels.

• According to international standards (e.g., ECDC), classification of XDR, and PDR requires: Testing across multiple antimicrobial classes and using validated methods for each drug class

2. To confidently classify isolates as XDR or PDR:

• Combine disk diffusion with: Broth microdilution, automated systems (e.g., VITEK, Phoenix) or gradient diffusion (E-test) for MIC determination

• Ensure the antibiotic panel covers all major antimicrobial categories relevant to the organism.

• The classification of XDR and PDR was based primarily on disk diffusion results, which may not fully capture resistance to all relevant antimicrobial categories, particularly for agents requiring MIC-based methods such as colistin. Therefore, the reported proportions of XDR and PDR should be interpreted with caution and requires revision.

• Lines 131 and 132: Remove ‘Blood culture samples collection, inoculation, identification and antimicrobial susceptibility testing

• Definitions (MDR, XDR and PDR) better be presented using bullet for easy of understanding than a paragraph

• How can you analyze the AST for MDR, XDR and PDR? Which tool used in this case?

• Avoid: This is table 2/3 legend.

• It is possible to assess the MDR, XDR or PDR for a single species, but it is totally wrong to determine/calculate the overall MDR/XDR/PDR for Gram-negatives or Gram-positives because we cannot use the same antimicrobials for all isolates. Therefore, this part should be reconsidered.

• Think of: ‘the Amhara National Regional State’ throughout the manuscript including table/figure titles.

• Why you consider the Addisalem General Hospital (AGH) as a reference variable the associated variables?

• If you mentioned some name of hospitals, why you left others? Were these the only hospitals that referred samples to APHI?

• The ‘discussion’ was too bulky (8 pages). It should be based on the study objective/s. It requires revision and to be targeted.

• Excessive numbers in the discussion part should be minimized

• Too many references for such a single objective and requires revision.

• Lines 19, 25 & 47: Reference, not Organization, W.H. Better say WHO.

• The titles of figures should be avoided from figures if they already placed in the main body of the manuscript. Titles should not be embedded in figures.

• Line 697: Reference (28); There was no article name, author, etc.

Reviewer #5: The revision includes meaningful improvements, particularly regarding contamination definitions and laboratory procedures. However, substantial methodological and analytical clarifications are still required.

Major Concerns

1. Extremely high blood culture positivity rate (~58%)

The reported culture positivity (58.3–58.5%) remains unusually high for routine retrospective laboratory data, even in referral laboratories.

Although the authors now describe contamination criteria (CDC/NHSN), important issues remain:

• Coagulase-negative staphylococci (CoNS) still represent a large proportion of isolates.

• The manuscript does not report:

o number of culture sets per patient actually analyzed

o proportion of isolates meeting “two consecutive cultures” requirement

o contamination rate

o proportion excluded as contaminants

Could the authors report the contamination rate according to their stated criteria?

Could the authors specify how many CoNS isolates were excluded vs retained as true pathogens?

I suggest that the authors provide a flow diagram showing:

• total cultures

• positive cultures

• contaminants excluded

• final pathogen count

Without this, the reported positivity rate remains difficult to interpret.

2. Identification methods remain limited

Bacterial identification relies entirely on conventional biochemical testing.

No confirmatory methods are reported (e.g., automated systems or advanced identification platforms).

While resource constraints are acknowledged, this limitation must be addressed analytically.

I suggest that the authors discuss the potential for species misidentification and its impact on resistance estimates.

Minor Concerns

Numerous grammatical and typographical errors remain.

Scientific nomenclature inconsistent (e.g., species formatting).

Some abbreviations are introduced without definition.

Tables are difficult to interpret due to inconsistent formatting.

Resistance percentages sometimes do not correspond clearly to denominators.

Background section contains repetitive information.

Several sentences require language editing for clarity.

**********

7. PLOS authors have the option to publish the peer review history of their article (what does this mean?). If published, this will include your full peer review and any attached files.

If you choose “no”, your identity will remain anonymous but your review may still be made public.

**Do you want your identity to be public for this peer review?**  For information about this choice, including consent withdrawal, please see our Privacy Policy.

Reviewer #2: No

Reviewer #5: No

**********

[NOTE: If reviewer comments were submitted as an attachment file, they will be attached to this email and accessible via the submission site. Please log into your account, locate the manuscript record, and check for the action link "View Attachments". If this link does not appear, there are no attachment files.]

To ensure your figures meet our technical requirements, please review our figure guidelines: https://journals.plos.org/plosone/s/figures

You may also use PLOS’s free figure tool, NAAS, to help you prepare publication quality figures: https://journals.plos.org/plosone/s/figures#loc-tools-for-figure-preparation.

NAAS will assess whether your figures meet our technical requirements by comparing each figure against our figure specifications.

# Author comment: Author response to Decision Letter 3

**DOI:** 10.1371/journal.pone.0354710.r006

24 Jun 2026

Dear Dr. Mengistu,

Academic Editor

PLOS ONE

Authors' response to reviewers' comments:

Reviewer #2:

The disk diffusion method (Kirby–Bauer) is useful for routine AST, but it has limitations when classifying advanced resistance categories like XDR and PDR:

Response: Thank you for taking the time to review our work and for raising such thoughtful and critical questions. To address these limitations, we have strict adherence to standard testing protocol, Standardized composition of the medium (MHA), Specimen-species-specific antimicrobial susceptibility testing and standardized antibiotic discs used to minimize the influence of variables. Additionally, we utilized recent CLSI guidelines for updating breakpoints and interpretation to improve the accuracy and reliability of AST results.

XDR and PDR definitions require testing against all or nearly all relevant antimicrobial categories.

Response: Dear reviewer, thank you so much for your comment. Multiple studies and standard protocols (CLSI) indicate that disc diffusion (Kirby-Bauer testing) for MDR/XDR/PDR determination typically involve 7–12 distinct antibiotics representing multiple classes: so that, we have tested 12 antibiotics for MDR/XDR/PDR classifications for antimicrobial susceptibility assessments.

β-lactams: ampicillin, amoxicillin-clavulanate, piperacillin-tazobactam, ceftriaxone, ceftazidime

Carbapenems: imipenem, meropenem

Aminoglycosides: gentamicin, tobramycin

Fluoroquinolones: ciprofloxacin

Others: trimethoprim/sulfamethoxazole, chloramphenicol

Disk diffusion panels often exclude critical last-resort drugs such as: colistin, tigecycline and new β-lactam/β-lactamase inhibitor combinations.

Response: Dear reviewer, thank you so much for your comment. Guidelines recommend AST is typically reserved for selected high-risk clinical isolates rather than included in routine AST panels. Therefore, routine AST for colistin in the study area was limited by technical complexity (labor-intensive, time-consuming, and required trained personnel), lack of reagents, high cost and limited routine clinical demand.

Disk diffusion is not reliable for some antibiotics (e.g., colistin), where broth microdilution is the gold standard.

Response: Dear Reviewer, thank you for your valuable comment. Several reports have mentioned colistin as the last-resort antibiotic used for treating multidrug-resistant Gram-negative infections. Even though we did not conduct and reported colistin resistance testing through disk diffusion method in the study area due to the following reasons technical complexity (labor-intensive, time-consuming, and require trained personnel), lack of reagents and high cost.

XDR/PDR classification often benefits from minimum inhibitory concentration (MIC) values to accurately determine resistance levels.

Response: Dear reviewer, thank you for your valuable comment. These classifications are crucial for understanding the severity of antibiotic resistance and guiding treatment strategies. However, initial detection or screening for MDR/XDR/PDR isolates before confirmatory MIC testing conducted through a widely used disk diffusion technique in our study area.

According to international standards (e.g., ECDC), classification of XDR, and PDR requires: Testing across multiple antimicrobial classes and using validated methods for each drug class

Response: Dear reviewer, thank you so much for your comment. Multiple studies (Sharma R, Otieno S, Otieno S. Kirby Bauer disc diffusion method for antibiotic susceptibility testing. Microbe Notes. 2022) and CLSI M100 standards indicate that disc diffusion (Kirby-Bauer testing) for MDR/XDR/PDR determination typically involve 7–12 relevant antibiotic classes, prevents underestimating resistance and ensures reliable classification into MDR/XDR/PDR. So, we have tested 12 antibiotics for XDR and PDR classifications for antimicrobial susceptibility assessments.

To confidently classify isolates as XDR or PDR:

Combine disk diffusion with: Broth microdilution, automated systems (e.g., VITEK, Phoenix) or gradient diffusion (E-test) for MIC determination.

Response: We sincerely thank you for your valuable comment. We agree that confidently classifying isolates as XDR or PDR ideally requires combining disk diffusion with additional methods such as broth microdilution, automated systems (e.g., VITEK, Phoenix), or gradient diffusion (E-test). Each of these methods has distinct advantages and limitations, and their selection depends on laboratory capacity and the bacterial species under investigation. However, in our study setting, limited accessibility and high costs restricted the use of these advanced methods. This constraint has been clearly acknowledged in the Limitations section of the manuscript.

Lines 131 and 132: Remove ‘Blood culture samples collection, inoculation, identification and antimicrobial susceptibility testing.

Response: Thank you for this insightful suggestion; we have acted on it by removing the paragraph.

Definitions (MDR, XDR and PDR) better be presented using bullet for easy understanding than a paragraph

Response: I truly appreciate your thoughtful feedback. Your observations provide meaningful guidance for enhancing the quality of this manuscript.

How can you analyze the AST for MDR, XDR and PDR? Which tool is used in this case?

Response: Analyzing Antibiotic Susceptibility Testing (AST) data to classify bacterial isolates as Multidrug-Resistant (MDR), Extensively Drug-Resistant (XDR), or Pandrug-Resistant (PDR) relies on evaluating resistance across standardized antimicrobial categories rather than individual drugs. According to the international consensus definitions established by the CDC and ECDC, an isolate is MDR if it is non-susceptible (resistant or intermediate) to at least one agent in three or more therapeutic categories. It is escalated to XDR if it remains susceptible to only one or two categories, and PDR if it is non-susceptible to every agent across all categories tested. To accurately perform this assessment, individual tested antibiotics must be mapped to their overarching classes (such as grouping Meropenem and Imipenem under Carbapenems), and a whole class is flagged as non-susceptible if even a single drug within it fails, while excluding any categories of natural, intrinsic resistance.

While raw AST data is initially generated in our laboratories through manual disk diffusion methods, WHONET software is the primary tool utilized to crunch these results into their proper resistance profiles and instantly flag MDR, XDR, and PDR isolates for public health reporting.

Avoid: This is table 2/3 legend.

Response: Dear reviewer, thank you so much for your comment. We have corrected table 2/3 legend based on your suggestion.

It is possible to assess the MDR, XDR or PDR for a single species, but it is totally wrong to determine/calculate the overall MDR/XDR/PDR for Gram-negatives or Gram-positives because we cannot use the same antimicrobials for all isolates. Therefore, this part should be reconsidered.

Response: thank you for your nice comments and suggestions. However, we can calculate Overall % prevalence as follows:

"MDR (%)"=N_"MDR" /N_"total" ×100

"XDR (%)"=N_"XDR" /N_"total" ×100

"PDR (%)"=N_"PDR" /N_"total" ×100

Think of: ‘the Amhara National Regional State’ throughout the manuscript including table/figure titles.

Response: I appreciate your insightful comments about the inclusion of Amhara Public Health Institute in the article, especially the titles of the tables and figures. We included it.

Why do you consider the Addisalem General Hospital (AGH) as a reference variable the associated variables?

Response: I really appreciate your feedback. Addisalem General Hospital (AGH) is considered a reference variable in these studies because of its significant involvement in patient-centered care and quality management strategies.

If you mentioned some names of hospitals, why did you leave others? Were these the only hospitals that referred samples to APHI?

Response: We appreciate the reviewer’s observation regarding the inclusions of some hospitals only and others left. The Amhara Public Health Institute (APHI) has faced challenges in receiving blood culture samples from hospitals due to limited referrals.

The ‘discussion’ was too bulky (8 pages). It should be based on the study objective/s. It requires revision and to be targeted.

Response: We acknowledge the reviewer’s concern regarding the bulkiness of the discussion. In response, we have:

• Reduced the length by eliminating non-essential details.

• Reorganized the content to follow the sequence of the study objectives.

• Strengthened the linkage between results and interpretation.

This targeted revision ensures that the discussion remains focused, coherent, and aligned with the purpose of the study

Excessive numbers in the discussion part should be minimized

Response: We acknowledge the reviewer’s concern regarding the bulkiness of the discussion.

Too many references for such a single objective and requires revision.

Response: We appreciate your observation regarding the number of references cited for this specific objective. We understand that excessive referencing may reduce clarity and conciseness. In response, we have carefully reviewed the cited literature and retained only the most relevant and recent sources that directly support the objective. This revision streamlines the reference list, ensuring that each citation adds clear value to the discussion without redundancy.

Lines 19, 25 & 47: Reference, not Organization, W.H. Better say WHO.

Response: Dear reviewer, I truly appreciate your feedback. We make amends for it.

The titles of figures should be avoided if they are already placed in the main body of the manuscript. Titles should not be embedded in figures.

Response: Dear reviewer, I really value your comments. we removed it.

Line 697: Reference (28); There was no article name, author, etc.

Response: We appreciate your comments. The article’s name, author, and other details have been added and updated in the reference (28).

Reviewer #5:

The revision includes meaningful improvements, particularly regarding contamination definitions and laboratory procedures. However, substantial methodological and analytical clarifications are still required.

Response: We appreciate the reviewer’s acknowledgment of the improvements made in defining contamination and refining laboratory procedures. We recognize the need for further methodological and analytical clarifications. In the revised manuscript, we have expanded the methodology section to provide clearer descriptions of sampling protocols, analytical techniques, and data interpretation steps.

The reported culture positivity (58.3–58.5%) remains unusually high for routine retrospective laboratory data, even in referral laboratories. Although the authors now describe contamination criteria (CDC/NHSN), important issues remain:

Coagulase-negative staphylococci (CoNS) still represent a large proportion of isolates.

The manuscript does not report number of culture sets per patient actually analyzed.

proportion of isolates meeting “two consecutive cultures” requirement

contamination rate

proportion excluded as contaminants

Response: We appreciate the reviewer’s comments and suggestions. Regarding the high reported culture positivity rate (58.3–58.5%), we carefully reviewed our methodology and data collection process. The observed rate may reflect specific factors, optimal blood volume, number of culture sets, laboratory practices and patients diagnosed with sepsis tend to have higher culture positivity rates. We have clarified these points in the revised manuscript and added a discussion regarding possible reasons for the higher positivity rate and its implications in the Context and Discussion sections. We believe these revisions adequately address the concerns raised and improve the clarity of our findings.

Although the authors now describe contamination criteria (CDC/NHSN), important issues remain:

Coagulase-negative staphylococci (CoNS) still represent a large proportion of isolates.

number of cultures sets per patient actually analyzed

proportion of isolates meeting “two consecutive cultures” requirement

contamination rate

proportion excluded as contaminants

Response: Various studies show that the contamination rate ranges between 0.6-6% (2-4); it depends upon the sample collection method and processing in the laboratory. The benchmark of blood contamination rate is <3%, as proposed by the Clinical Laboratory Standard Institute (CLSI) …

Could the authors report the contamination rate according to their stated criteria?

Response: Yes, the authors can report the contamination rate in accordance with their defined criteria.

Could the authors specify how many CoNS isolates were excluded vs retained as true pathogens? But the report must: By following these steps, the manuscript will meet standard expectations for reproducibility and transparency in microbial contamination reporting

Clearly define sample eligibility and contaminant classification.

Use explicit calculation methods with numerators and denominators stated.

Provide statistical confidence intervals.

Disclose any sampling or decontamination procedures influencing results.

Response: Based on the data provided in the referenced studies, the number of Coagulase-negative Staphylococci (CoNS) isolates retained as true pathogens versus those excluded as contaminants is reported as follows:

Total 817 blood culture records retrieved

58.9% (N= 481) yielded growth

Excluded (likely contaminants): 29.3 % (N=141) including Coagulase-negative staphylococci (CoNS) (27 %, N=130), Bacillus spp. (1.2 %, N= 6) and Candida spp. (1 %, N= 5).

Remaining 676 blood culture records were included in the final analysis

True pathogens: 50.3 % (N=340)

2.21. Identification methods remain limited

Bacterial identification relies entirely on conventional biochemical testing.

No confirmatory methods are reported (e.g., automated systems or advanced identification platforms).

While resource constraints are acknowledged, this limitation must be addressed analytically.

I suggest that the authors discuss the potential for species misidentification and its impact on resistance estimates.

Response: Despite the advent of many newer tests, biochemical tests are still pivotal for bacterial identification. Phenotypic and biochemical tests still hold the key for laboratory identification due to their lower cost and ease in carrying out and interpretation. Biochemical tests are the key for easily microbial identification for researchers in past, present and also in future, from various sites of infections.

Numerous grammatical and typographical errors remain.

Response: Thank the reviewer for your constructive comments and the time you spent reviewing this manuscript. These improvements are made through your constructive feedback.

Scientific nomenclature inconsistent (e.g., species formatting).

Response: Thank the reviewer for your constructive comments and we amend it.

Some abbreviations are introduced without definition.

Response: Thank you for pointing this out. The abbreviations have been included with their definitions where the change can be found in the revised manuscript.

Tables are difficult to interpret due to inconsistent formatting.

Response: We think this is an excellent suggestion. We have followed consistent formatting thought the Table and the change can be found in the revised manuscript.

Resistance percentages sometimes do not correspond clearly to denominators.

Response: Thank you for pointing this out. We have revised it.

Background section contains repetitive information.

Response: We appreciate the reviewer’s observation regarding the repetition in the background section. We have carefully revised this section to remove redundant statements and streamline the narrative. Overlapping information has been consolidated, and each paragraph now focuses on a distinct aspect of the study’s context to ensure clarity and conciseness. These changes improve the flow and avoid unnecessary reiteration while retaining essen

Attachment Submitted filename: Response_to_Reviewers_auresp_3.docx

# Editor report: Decision Letter 3

**DOI:** 10.1371/journal.pone.0354710.r007

**Authors:** Mengistu Zenebe

12 Jul 2026

Bacterial profile and antimicrobial resistance patterns of bloodstream infections at Amhara Public Health Institute, Bahir Dar, Ethiopia

PONE-D-25-16842R3

Dear Dr. Getie,

We’re pleased to inform you that your manuscript has been judged scientifically suitable for publication and will be formally accepted for publication once it meets all outstanding technical requirements.

Within one week, you’ll receive an e-mail detailing the required amendments. When these have been addressed, you’ll receive a formal acceptance letter and your manuscript will be scheduled for publication.

An invoice will be generated when your article is formally accepted. Please note, if your institution has a publishing partnership with PLOS and your article meets the relevant criteria, all or part of your publication costs will be covered. Please make sure your user information is up-to-date by logging into Editorial Manager at Editorial Manager® and clicking the ‘Update My Information' link at the top of the page. For questions related to billing, please contact billing support.

If your institution or institutions have a press office, please notify them about your upcoming paper to help maximize its impact. If they’ll be preparing press materials, please inform our press team as soon as possible -- no later than 48 hours after receiving the formal acceptance. Your manuscript will remain under strict press embargo until 2 pm Eastern Time on the date of publication. For more information, please contact onepress@plos.org.

Kind regards,

Mengistu Hailemariam Zenebe, PhD

Academic Editor

PLOS One

Additional Editor Comments (optional):

Reviewers' comments:

# Editor report: Acceptance letter

**DOI:** 10.1371/journal.pone.0354710.r008

**Authors:** Mengistu Zenebe

PONE-D-25-16842R3

PLOS One

Dear Dr. Getie,

I'm pleased to inform you that your manuscript has been deemed suitable for publication in PLOS One. Congratulations! Your manuscript is now being handed over to our production team.

At this stage, our production department will prepare your paper for publication. This includes ensuring the following:

* All references, tables, and figures are properly cited

* All relevant supporting information is included in the manuscript submission,

* There are no issues that prevent the paper from being properly typeset

You will receive further instructions from the production team, including instructions on how to review your proof when it is ready. Please keep in mind that we are working through a large volume of accepted articles, so please give us a few days to review your paper and let you know the next and final steps.

Lastly, if your institution or institutions have a press office, please let them know about your upcoming paper now to help maximize its impact. If they'll be preparing press materials, please inform our press team within the next 48 hours. Your manuscript will remain under strict press embargo until 2 pm Eastern Time on the date of publication. For more information, please contact onepress@plos.org.

You will receive an invoice from PLOS for your publication fee after your manuscript has reached the completed accept phase. If you receive an email requesting payment before acceptance or for any other service, this may be a phishing scheme. Learn how to identify phishing emails and protect your accounts at https://explore.plos.org/phishing.

If we can help with anything else, please email us at customercare@plos.org.

Thank you for submitting your work to PLOS ONE and supporting open access.

Kind regards,

PLOS ONE Editorial Office Staff

on behalf of

Dr. Mengistu Hailemariam Zenebe

Academic Editor

PLOS One
